# Pathogen-specific structural features of *Candida albicans* Ras1 activation complex: uncovering new antifungal drug targets

**DOI:** 10.1128/mbio.00638-23

**Published:** 2023-08-01

**Authors:** José A. Manso, Arturo Carabias, Zsuzsa Sárkány, José M. de Pereda, Pedro José Barbosa Pereira, Sandra Macedo-Ribeiro

**Affiliations:** 1 IBMC–Instituto de Biologia Molecular e Celular, Universidade do Porto, Porto, Portugal; 2 i3S–Instituto de Investigação e Inovação em Saúde, Universidade do Porto, Porto, Portugal; 3 Instituto de Biología Molecular y Celular del Cáncer, Consejo Superior de Investigaciones Científicas-University of Salamanca, Salamanca, Spain; Columbia University, New York, New York, USA

**Keywords:** Ras-like protein 1, cell division control protein 25, X-ray crystallography, small angle X-ray scattering, polyQ

## Abstract

An important feature associated with *Candida albicans* pathogenicity is its ability to switch between yeast and hyphal forms, a process in which CaRas1 plays a key role. CaRas1 is activated by the guanine nucleotide exchange factor (GEF) CaCdc25, triggering hyphal growth-related signaling pathways through its conserved GTP-binding (G)-domain. An important function in hyphal growth has also been proposed for the long hypervariable region downstream the G-domain, whose unusual content of polyglutamine stretches and Q/N repeats make CaRas1 unique within Ras proteins. Despite its biological importance, both the structure of CaRas1 and the molecular basis of its activation by CaCdc25 remain unexplored. Here, we show that CaRas1 has an elongated shape and limited conformational flexibility and that its hypervariable region contains helical structural elements, likely forming an intramolecular coiled-coil. Functional assays disclosed that CaRas1-activation by CaCdc25 is highly efficient, with activities up to 2,000-fold higher than reported for human GEFs. The crystal structure of the CaCdc25 catalytic region revealed an active conformation for the α-helical hairpin, critical for CaRas1-activation, unveiling a specific region exclusive to CTG-clade species. Structural studies on CaRas1/CaCdc25 complexes also revealed an interaction surface clearly distinct from that of homologous human complexes. Furthermore, we identified an inhibitory synthetic peptide, prompting the proposal of a key regulatory mechanism for CaCdc25. To our knowledge, this is the first report of specific inhibition of the CaRas1-activation via targeting its GEF. This, together with their unique pathogen-structural features, disclose a set of novel strategies to specifically block this important virulence-related mechanism.

**IMPORTANCE:**

*Candida albicans* is the main causative agent of candidiasis, the commonest fungal infection in humans. The eukaryotic nature of *C. albicans* and the rapid emergence of antifungal resistance raise the challenge of identifying novel drug targets to battle this prevalent and life-threatening disease. CaRas1 and CaCdc25 are key players in the activation of signaling pathways triggering multiple virulence traits, including the yeast-to-hypha interconversion. The structural similarity of the conserved G-domain of CaRas1 to those of human homologs and the lack of structural information on CaCdc25 has impeded progress in targeting these proteins. The unique structural and functional features for CaRas1 and CaCdc25 presented here, together with the identification of a synthetic peptide capable of specifically inhibiting the GEF activity of CaCdc25, open new possibilities to uncover new antifungal drug targets against *C. albicans* virulence.

## INTRODUCTION

*Candida albicans*, part of the commensal microbiota of most individuals, is an important opportunistic human fungal pathogen and can cause severe infections in immunocompromised patients (1). *C. albicans* belongs to the CTG-clade of *Ascomycetes*, which includes a high number of opportunistic pathogenic species that use an alternative genetic code and translate the CUG codon as a serine instead of leucine (2, 3). The remarkable adaptability of *C. albicans* to highly diverse host niches (4) is underscored by its phenotypic plasticity, i.e., the discrete phenotypes adopted in response to varying environmental cues (5). The World Health Organization (WHO), in a recent report (fungal priority pathogens list, WHO-FPPL) classified *C. albicans* as a first-priority pathogen and member of the critical group (6), highlighting the emergency in the discovery and development of novel drugs to battle this important human pathogen. The commensal-to-pathogen transition of *C. albicans* is associated with its ability to interconvert between yeast and hyphal morphologies (7 - 9). The activation of hyphae-specific genes is directly mediated by the cyclic adenosine monophosphate (cAMP) and the mitogen-activated protein kinase (MAPK) signaling pathways, which are regulated by the Ras-like protein 1 (CaRas1) and the cell division control protein 25 (CaCdc25) (10, 11).

CaRas1 is composed of a highly conserved GTPase domain (G-domain) followed by a hypervariable region. The long hypervariable region of CaRas1 represents a major difference between yeast (~120 residues) and human (~20 residues) Ras proteins. Further, the presence of low complexity segments in the hypervariable region of CaRas1, including polyglutamine (PolyQ) repeats and a Q/N-rich stretch, also present in other members of the CTG-clade, makes it unique among Ras-family proteins (Fig. S1). The hypervariable region of CaRas1 also contains the conserved C-terminal CCaaX membrane association motif, whose two cysteine residues have been found to be farnesylated and palmitoylated (12), in agreement with the localization of CaRas1 to membranes, as observed for many Ras GTPases. Interestingly, cleavage of CaRas1 at its hypervariable region, removing the last 67 amino acid residues, has been proposed as a mechanism modulating CaRas1 signaling and, consequently, hyphal growth (13).

In yeast cells, CaRas1 is generally in an inactivated, GDP-bound form (CaRas1-GDP). CaCdc25 activates CaRas1, promoting its conversion to CaRas1-GTP and switching on the signaling pathway associated with the hyphae-specific genes (11, 14, 15). The activation of CaRas1 by CaCdc25 is the primary mechanism by which D-glucose induces morphogenesis (11, 15, 16). In fact, deletion of either CaRas1 or CaCdc25 results in hyphal defects (17). CaCdc25 (~150 kDa) is a Ras-guanine nucleotide exchange factor (GEF) that contains a C-terminal catalytic region, consisting of the tandem Ras-exchange motif domain-catalytic domain (REM-CAT), which catalyzes the exchange of CaRas1-bound GDP to GTP.

Despite its biological relevance, no experimental structural information is available for the full-length CaRas1 and the relative arrangement of the G-domain and the unique low complexity hypervariable region, which is predicted to be partially disordered. Also, the elucidation of the molecular basis for the activation of CaRas1 by CaCdc25 would provide crucial information for the design of new drugs to fight this fungal human pathogen.

In this work, by combining biophysical techniques with artificial intelligence (AI)-based predictive modeling, we disclose structural features of this unique Ras-family protein and of its GEF CaCdc25, both isolated and complexed. We also identify a synthetic peptide capable of inhibiting the activity of CaCdc25, shedding light on its mechanism of regulation and establishing a path for the rational design of novel inhibitors of this fungal signaling pathway.

## RESULTS

### The domain organization and structure of CaRas1 are unique among Ras-like proteins

**Fig 1:**
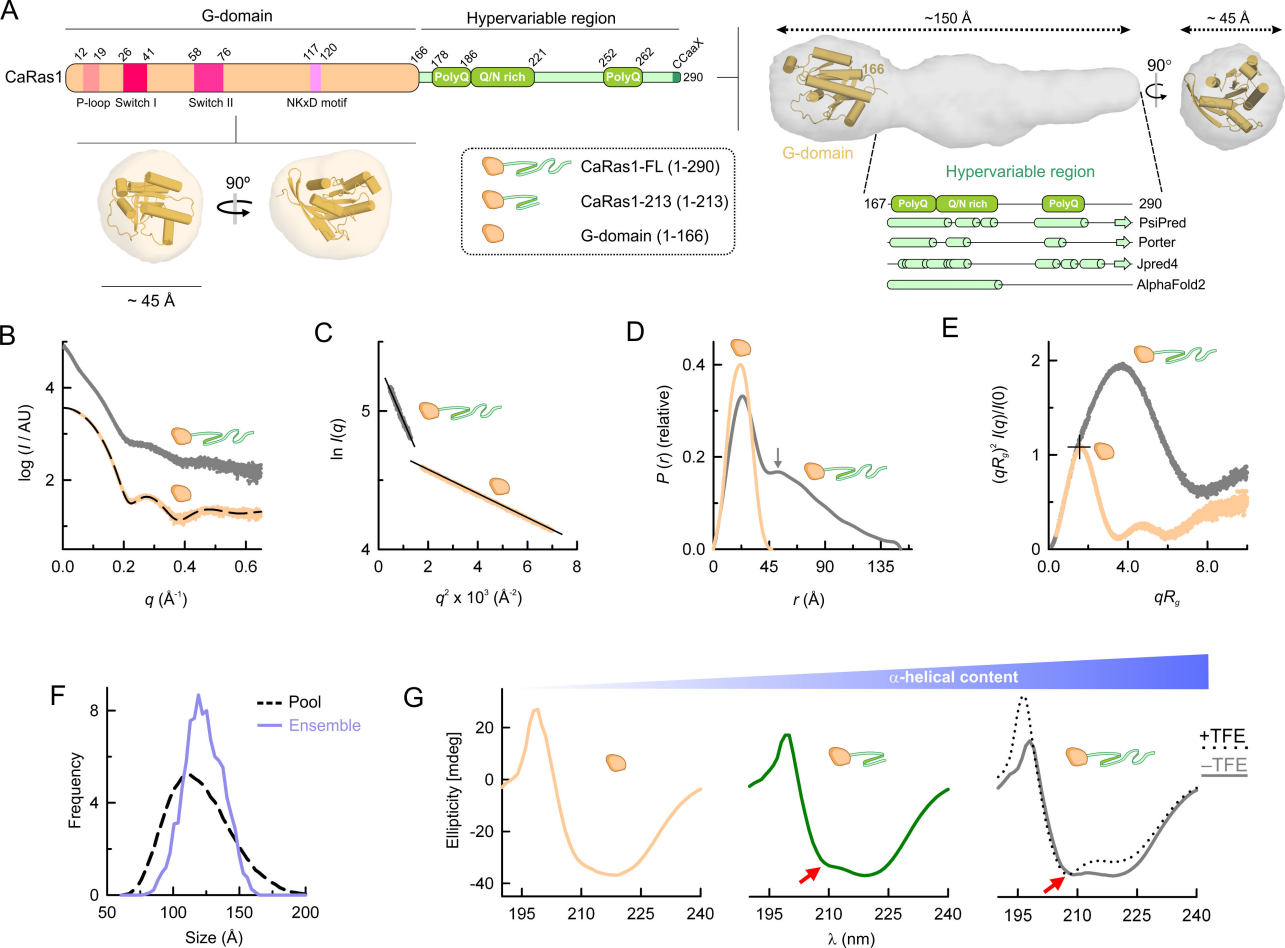
The hypervariable region of CaRas1 displays an unexpected architecture. (**A**) Domain organization scheme for CaRas1, highlighting the G-domain (residues 1–166) and the hypervariable region (residues 167–290). Important structural elements of the G-domain—nucleotide exchange P-loop, Switches I and II, and NKxD motif—and the polyQ and Q/N-rich segments within the hypervariable region are labeled. Schemes of the different constructs used in this work are provided in the dotted box. The SAXS-derived molecular envelopes for the CaRas1 G-domain and for full-length CaRas1 are shown as semi-transparent surfaces, below and to the right of the domain organization scheme, respectively. A cartoon representation (orange) of the G-domain model is docked into the envelopes. Below the envelope of full-length CaRas1, predicted secondary structural elements for the hypervariable region are represented, suggesting that the polyQ and Q/N-rich regions are mainly helical. (**B**) Experimental SAXS profiles extrapolated to infinite dilution of CaRas1-FL (dark-gray) and G-domain (orange). Curves are offset on the log scale for readability; the same data color scheme is used in panels (C–G). The scattering calculated for the atomic model of the G-domain shown in panel A is represented as a black dashed line. (**C**) Guinier plots of the scattering data shown in B. (**D**) *P*(*r*) functions determined from the scattering data shown in B. The maximum for a second peak in CaRas1-FL is indicated by a gray arrow. (**E**) Dimensionless Kratky plots. The crosshair indicates the expected position of the theoretical maximum of the plot for spherical compact particles (*qR*_g_ ~1.732 and (*qR*_g_)^2^*I*(*q*)/*I*(0) ~1.104). (**F**) Ensemble optimization methods (EOM) analysis of the flexibility of the hypervariable region. Frequency of size distributions in a pool of 10,000 models (black dashed line) with random orientations of the hypervariable region, and in the selected ensemble that fits the SAXS data of CaRas1-FL (blue line). (**G**) Circular dichroism spectra of CaRas1 G-domain, CaRas1-213 (green), and CaRas1-FL. The negative band at 209 nm (red arrows) observed when the hypervariable region is present in CaRas1, together with that at 220 nm are spectral signatures for α-helical content. An inversion of the 220 nm/209 nm ratio (from 1.00 to 0.88) in the CD spectrum of CaRas1-FL was observed in the presence of 50% (vol/vol) TFE (dotted black line).

CaRas1 is composed by a highly conserved G-domain, followed by an exclusive hypervariable region that contains low complexity sequence segments, including PolyQ repeats and a Q/N-rich stretch (Fig. 1A), a unique feature conserved in Ras-family proteins from various opportunistic fungal pathogens of the CTG-clade. The hypervariable region of CaRas1 is annotated as disordered in the MobiDB database (18), which incorporates consensus analysis from several disorder prediction servers. Interestingly, three independent secondary structure predictors—PsiPred, Porter, and JPred4—assign helical elements within the polyQ and Q/N rich stretches of the hypervariable region (Fig. 1A), in line with the predictions from AlphaFold2 (19). This is expected since there is extensive evidence in the literature supporting the idea that polyQ and Q-rich sequences have the ability to form α-helical and coiled-coil structures (20 - 24). This tendency is especially pronounced when these sequences are preceded by a region with high propensity for forming helical structures.

The structural properties of the hypervariable region within CaRas1 and its relative position with respect to the globular G-domain were evaluated using SAXS (Fig. 1A through F; Table S1) and two CaRas1 constructs, the full-length protein (CaRas1-FL, 1–290) and the isolated G-domain (1–166). Both CaRas1 constructs were monodisperse and monomeric in solution (Fig. 1C; Table S1). The distance distribution function [*P*(*r*)] for the G-domain has a bell shape characteristic of globular particles with a peak at ~23 Å and a maximum dimension (*D*_max_) of ~45 Å (Fig. 1D), in good agreement with a homology model for the G-domain (Fig. S2; Fig. 1A and B) that could be easily docked in a low-resolution envelope obtained by *ab initio* methods from the experimental SAXS data (Fig. 1A). Interestingly, the high resolution of the SAXS data allowed to distinguish between closely related homology models and suggests that the highly dynamic G-domain switch II region displays an α-helical structure in CaRas1 (Fig. S2). When the hypervariable region of CaRas1 is present, the *P*(*r*) function displays a second peak at ~52 Å (Fig. 1D), suggesting a certain degree of rigidity and the presence of structured elements in that region. In agreement, the low-resolution envelope for CaRas1-FL, which is an average of 15 independent *ab initio* models (Table S1), displays an elongated shape (Fig. 1A). The homology model for the G-domain could be docked in one end of this envelope, allowing the assignment of an adjacent volume to the hypervariable region (Fig. 1A). The differences between CaRas1-FL and its isolated G-domain are further highlighted in the dimensionless Kratky plot of the scattering data (25), which displays a bell-shaped peak with a maximum at 1.1 for the G-domain, indicating that it is a compact and spherical particle (Fig. 1E). When the hypervariable region is present, the overall shape of this plot is preserved, however the position and amplitude of its maximum deviates largely from the value expected for spherical particles, in line with the highly anisometric shape of CaRas1-FL (26) (Fig. 1E). The overall shapes of the bimodal *P*(*r*) function and the dimensionless Kratky plot for CaRas1-FL, are characteristic of a protein composed by relatively rigid two-bodies (27), suggesting that the hypervariable region is not entirely disordered.

In order to better understand the structure and flexibility of the hypervariable region, an ensemble fitting method was employed (28). A pool of 10,000 CaRas1-FL structures was generated, assuming full flexibility of the hypervariable region and sampling exhaustively its potential orientation. The minimal ensemble that better approximated the experimental SAXS data were mainly populated by conformations with a narrower size distribution than the entire size range of the pool (Fig. 1F), further underscoring the limited flexibility of the hypervariable region and in line with the calculated *R*sigma value of 0.61 for this analysis, as values <1.0 for this parameter are indicative of limited flexibility in the system. In agreement, circular dichroism (CD) analysis of the secondary structure of three CaRas1 truncation constructs with variable lengths of the hypervariable region (Fig. 1A) revealed an increase in α-helical content when the hypervariable region was present (Fig. 1G). Importantly, the CD spectrum of CaRas1-FL displayed minima at 220 nm and 209 nm, with a 220 nm/209 nm ratio of 1.00, supporting the presence of a coiled-coil structure in the hypervariable region. In the presence of 50% (vol/vol) trifluoroethanol (TFE), a strong α-helix stabilizer that disrupts coiled-coil formation, the 220 nm/209 nm ratio shifted to 0.88 (Fig. 1G), which is suggestive of a coiled-coil to isolated helices transition (29).

**Fig 2:**
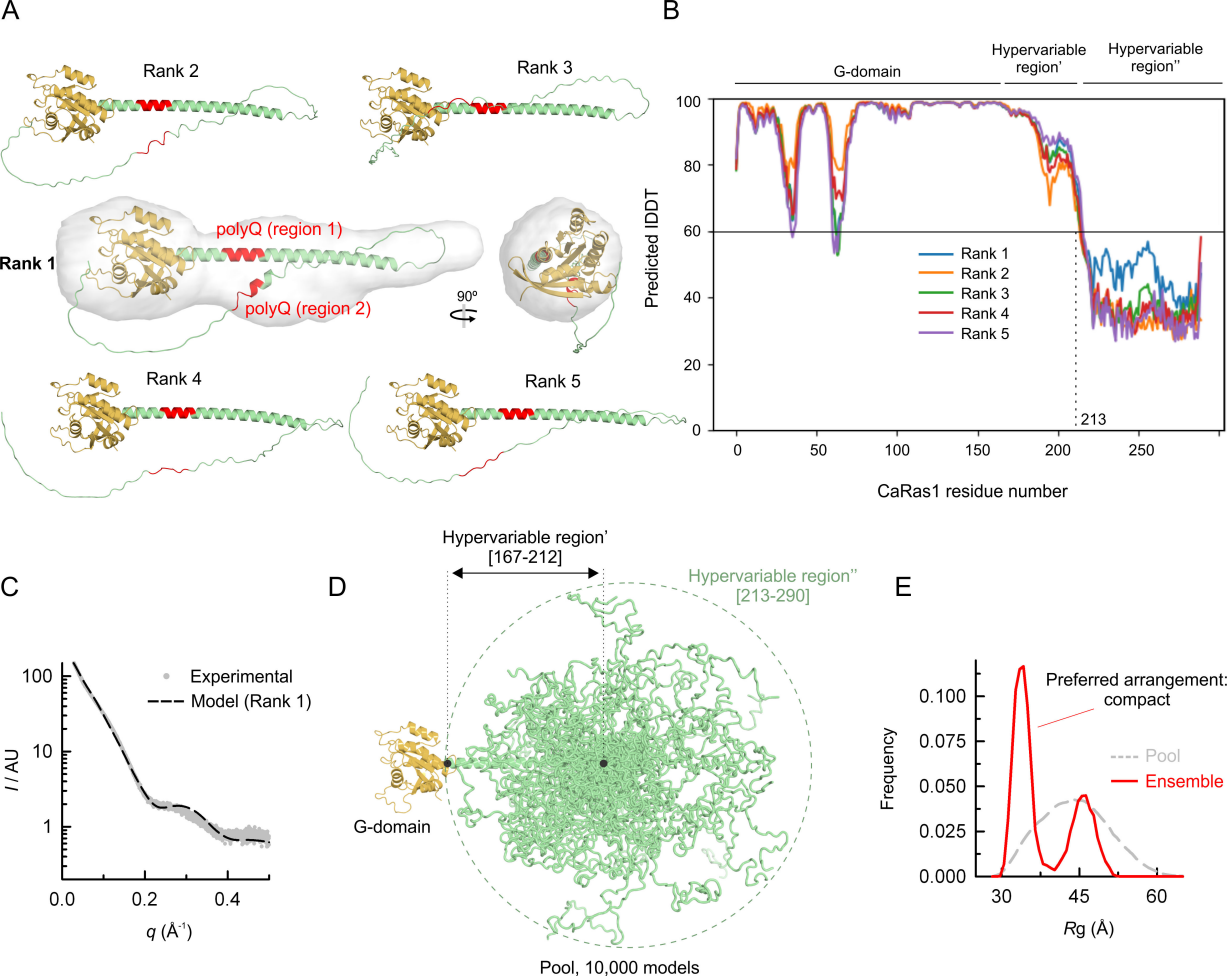
Structure of full-length CaRas1 and flexibility analysis of the hypervariable region segment 213–290. (**A**) The five top-ranking structural models predicted by AlphaFold2 for full-length CaRas1. The best model (ranked 1) is docked into the low-resolution experimental envelope. The two polyQ regions are highlighted in red. (**B**) Per-residue local Distance Difference Test (lDDT) of the five predicted structures. The segment 213–290 was predicted with low confidence (lDDT <60). (**C**) SAXS experimental data of full-length CaRas1 (gray dots) superposed with the scattering profile calculated for the best predicted model (dashed black line). (**D**) Representation of the pool of structures generated to evaluate by EOM analysis the flexibility of the CaRas1 region 213–290 (green). The pool consisted of 10,000 models, in which both the G-domain (orange) and the 167–212 region (green) were fixed, whereas the part of the hypervariable region comprising residues 213–290 was considered fully flexible (for clarity only 50 models are represented). (**E**) EOM analysis of the flexibility of the 213–290 region (hypervariable region"). Frequency of radius of gyration (*R*g) distributions in a pool of 10,000 models (gray dashed line) with random orientations of the hypervariable region", and in the selected ensemble that fits the experimental SAXS data of CaRas1-FL. Default parameters were employed using native-like models, allowing constant subtraction (0.149) and curve repetition (both the minimum number of curves per ensemble and the number of obtained representative structures, was five). The values for *R*flex(random)/*R*sigma of ~73.56% (~89.13%) / 0.91 indicate limited flexibility.

With the support of these experimental data, we explored further the predicted structure of full-length CaRas1 using the AI-based network AlphaFold2 (Fig. 2A through C). We generated five AlphaFold2 models, which predicted with high confidence (per-residue local Distance Difference Test, lDDT >70) an extension of the terminal helix of the G-domain through the first polyQ region until near the end of the Q/N stretch (N212). Interestingly, in some of the models obtained, AlphaFold2 predicted a helical component in the second polyQ region and positioned it close to the first polyQ region, in line with the coiled-coil structure observed by CD. Importantly, the calculated SAXS profile of this model is quite similar to that obtained experimentally (Fig. 2C). Since the C-terminal part of the hypervariable region (residues 213–290) was predicted with low confidence (lDDT <60), the flexibility of this region was further validated by SAXS (Fig. 2D and E). Indeed, ensemble optimization methods (EOM) analysis of the subset of models that better fit the experimental SAXS profile underscored the preference for a compact arrangement.

### Structure of the catalytic region of CaCdc25 reveals a α-helical hairpin in an active conformation

**Fig 3:**
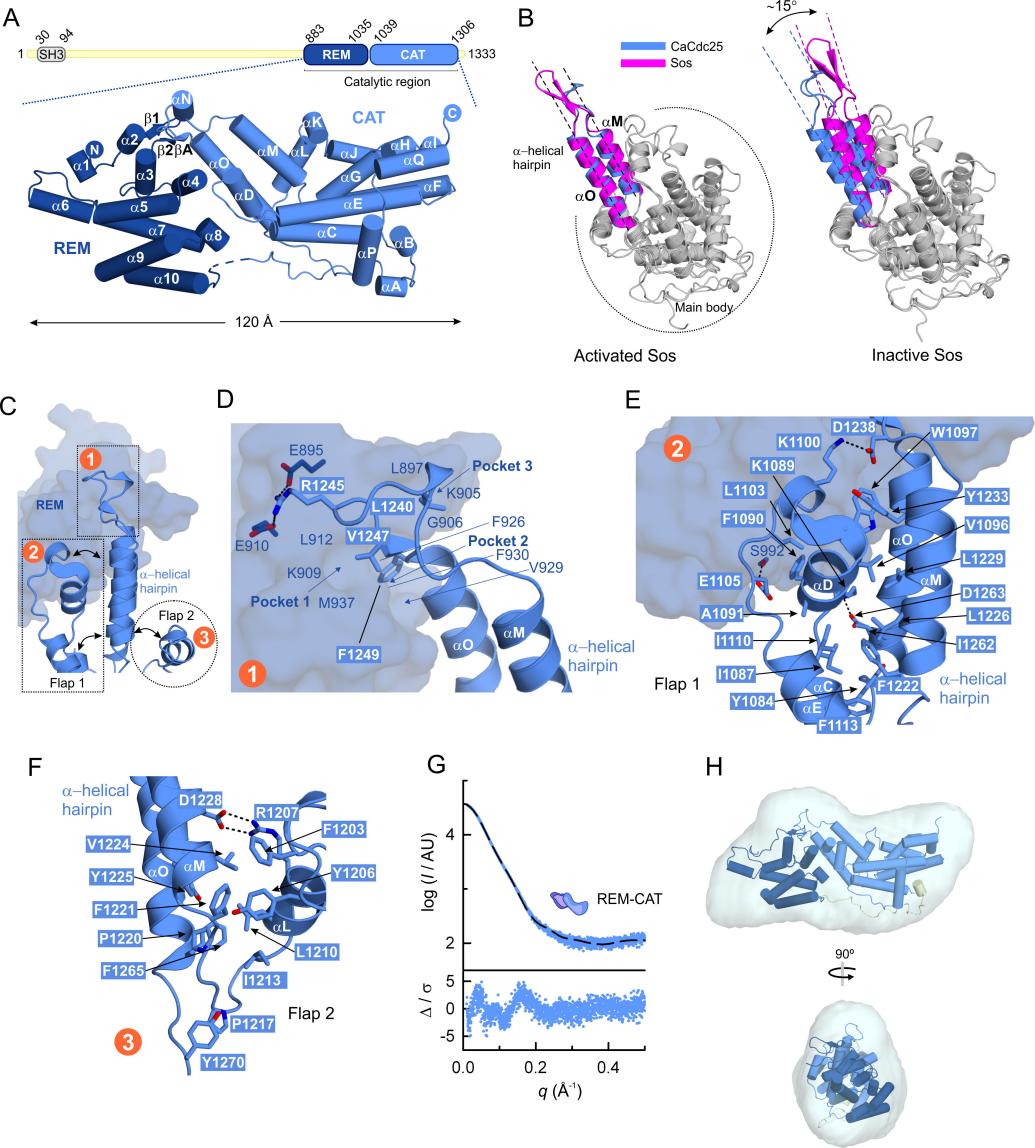
Structure of the catalytic region of CaRas1-guanine nucleotide exchange factor CaCdc25. (**A**) Domain organization diagram (top) and cartoon representation (bottom) of the crystal structure of the catalytic region of CaCdc25 at 2.45 Å resolution. The REM (dark blue) and CAT (light blue) domains are labeled and the N- and C- termini are indicated in filled circles. (**B**) Structural superposition of the main body of the CAT domain of CaCdc25 with the active [PDB entry 1NVW (31)] and inactive [PDB entry 2II0 (32)] forms of Sos showing that the α-helical hairpin in the CaCdc25 structure is poised for activity. (**C**) Overview of the three main interacting regions (numbered in orange circles) that maintain the α-helical hairpin in an activated position, detailed in panels D-F. (**D**) Interaction of the loop connecting the two main helices of the α-helical hairpin (main interacting residues shown as sticks) with the REM domain (transparent surface). (**E**) Interaction of the α-helical hairpin with the flap 1 region (main interacting residues shown as sticks) and with the REM domain (transparent surface). (**F**) Interaction of the α-helical hairpin with the flap 2 region (main interacting residues shown as sticks). (**G**) Experimental SAXS profile of the catalytic region of CaCdc25, extrapolated to infinite dilution (upper plot). The scattering calculated for the atomic structure of the tandem REM-CAT of CaCdc25, shown in A, is represented as a black dashed line. Error-weighted residual difference plot Δ/σ = [*I*_exp_(*q*) - *cI*_mod_(*q*)]/ σ (*q*) versus *q* (lower plot). (**H**) Crystal structure of CaCdc25 (cartoon representation) docked into the SAXS-derived molecular envelope (translucent surface), which is the average of 15 independent bead models. Two orthogonal views are shown.

The crystal structure of the catalytic region of the CaRas1 guanine nucleotide exchange factor CaCdc25, which catalyzes the exchange of the bound nucleotide and therefore the conversion of CaRas1-GDP to CaRas1-GTP, was determined at 2.45 Å-resolution (Fig. 3A through F; Table 1). The protein crystallized in the orthorhombic space group *P*2_1_2_1_2_1_ with two molecules of CaCdc25 in the asymmetric unit (AU) that are very similar (Fig. S3A and B). The complete monomers superpose with a root mean square deviation (rmsd) of 1.0 Å for 384 aligned *C*α atoms. A structural homology search with DALI (30) showed that, despite low amino acid sequence conservation, the catalytic region of CaCdc25 is structurally quite similar to human GEFs containing Cdc25 homology domains and that act on the Ras family (four matches with Z score >20; Fig. S3C), and consists of two well-differentiated domains: the REM domain (residues 883–1035, β-strands β**1** and β**2** and α-helices α**1**–α**10**) and the CAT domain (residues 1039–1306, β-strands β**A** and α-helices α**A**-α**Q**) (Fig. 3A). In particular, the individual structural elements for the CAT domain are more conserved in the five molecules (rmsd 3.7–4.7 Å for 286 aligned *C*α atoms) than those for the REM domain (rmsd 6.3–7.9 Å for 179 aligned *C*α atoms; Fig. S3D).

**TABLE 1:** Crystallographic data collection and refinement statistics^*a*^

	CaRas1 guanine-nucleotide exchange factor CaCdc25 (REM and CAT domains)	
	Data set A	Data set B
**Data collection**		
X-ray beamline	BL13-XALOC (ALBA)	
Space group	*P*2_1_2_1_2_1_	
Cell dimensions	*a* = 49.1 Å *b* = 107.8 Å *c* = 197.7 Å	*a* = 48.6 Å *b* = 107.2 Å *c* = 196.9 Å
Wavelength (Å)	0.97915	0.97926
Resolution range (Å)	98.84–3.00 (3.08–3.00)	43.22–2.45 (2.50–2.45)
Total/unique reflections	181,241/21,838 (19,494/2,154)	190,781/38,604 (9,513/1,803)
Average multiplicity	8.3 (9.1)	4.9 (5.0)
Completeness (%)	99.8 (100.0)	99.9 (100.0)
R meas^*b*^ (%)	51.3 (305.7)	16.5 (168.5)
R pim^*c*^ (%)	18.0 (98.2)	7.3 (75.3)
CC_1/2_ (%)	96.9 (41.0)	99.5 (45.4)
Mean *I*/σ*I*	7.0 (1.3)	8.5 (1.2)
**Refinement**		
Resolution range (Å)	47.68–3.0	43.22–2.45
Unique reflections, work/free	21,833/1,068	38,543/1,953
R work (%)	30.9	19.4
R free^*d*^ (%)	34.0	24.0
Number of:		
Amino acid residues	799	840
Water molecules	−	144
Average B value (Å^2^)		
Wilson plot	72.2	45.2
Protein	68.8	54.6
Solvent	−	52.4
rmsd bond lengths (Å)	0.003	0.002
rmsd bond angles (°)	0.67	0.45
Ramachandran		
Favored (%)	82.8	98.2
Allowed (%)	10.6	1.8
Outliers (%)	6.6	0
Rotamer outliers (%)	15.5	0
Clashscore	8.8	2.2
PDB entry	–	7NZZ
SBGrid Data Bank entry	doi:10.15785/SBGRID/860 doi:10.15785/SBGRID/861	doi:10.15785/SBGRID/859

^*a*^ Values in parenthesis correspond to the outermost resolution shell.

^*b*^ R meas is the multiplicity independent R factor.

^*c*^ R pim is the precision-indicating merging R factor.

^*d*^ Calculated using 5% of reflections that were not included in the refinement.

The α-helical hairpin of the CAT domains is a key element for the Ras nucleotide exchange in GEFs (33). In CaCdc25, the α-helical hairpin is formed by the two antiparallel helices α**M** and α**O** (residues 1222–1265) (Fig. 3B), which protrude from the main body of the CAT domain. The relative orientation of the α-helical hairpin is critical for the activity of GEFs. In CaCdc25, the hairpin assumes an orientation similar to that observed in the structure of the active state of the human HRas GEF Sos (Fig. 3B), suggesting that the CAT domain of CaCdc25 is also in the active conformation in the crystal structure. The active state-like orientation of the α-helical hairpin of CaCdc25 is stabilized mostly by hydrophobic interactions with three regions (Fig. 3C): (i) the REM domain, where α-helical hairpin residues L1240, V1247, and F1249 occupy three hydrophobic grooves formed by K909, L912, and M937 (pocket 1); F926, V929, and F930 (pocket 2); and L897, K905, and G906 (pocket 3) (Fig. 3D), (ii) a region called flap 1 (residues 1080–1115) that encompasses helix α**D** and part of helices α**C** and α**E** and that stabilizes helix α**M** of the hairpin (Fig. 3E), and (iii) the region called flap 2 (residues 1195–1215), which includes part of helix α**L** (Fig. 3F). In addition to these hydrophobic interactions, typical of the GEF family, the α-helical hairpin of CaCdc25 is further stabilized by three unique polar contacts, which are not observed in the structures of the most similar GEFs (Fig. S3C): R1245 interacts with E895 and E910 from the REM domain (Fig. 3D); D1238 interacts with K1100 from the flap 1 region; and E1105 from flap 1 interacts with S992 from the REM domain (Fig. 3E). Importantly, a multiple amino acid sequence alignment with human GEFs revealed only partial conservation of the residues mediating the activation of Ras in CaCdc25 (Fig. S4A). The two important residues located in the α-helical hairpin that impede nucleotide binding in the human GEF Sos1, L938, and E942 are replaced in CaCdc25 by T1230 and H1234, respectively.

Finally, the solution structure of the catalytic region of CaCdc25 is in excellent agreement with the crystallographic model (Fig. 3G; Table S1), which unsurprisingly docks well into the *ab initio* molecular envelope derived from the SAXS data (Fig. 3H).

### Negatively charged substitutions at the CTG-clade specific H1234 of the α-helical hairpin decrease the activity of CaCdc25

**Fig 4:**
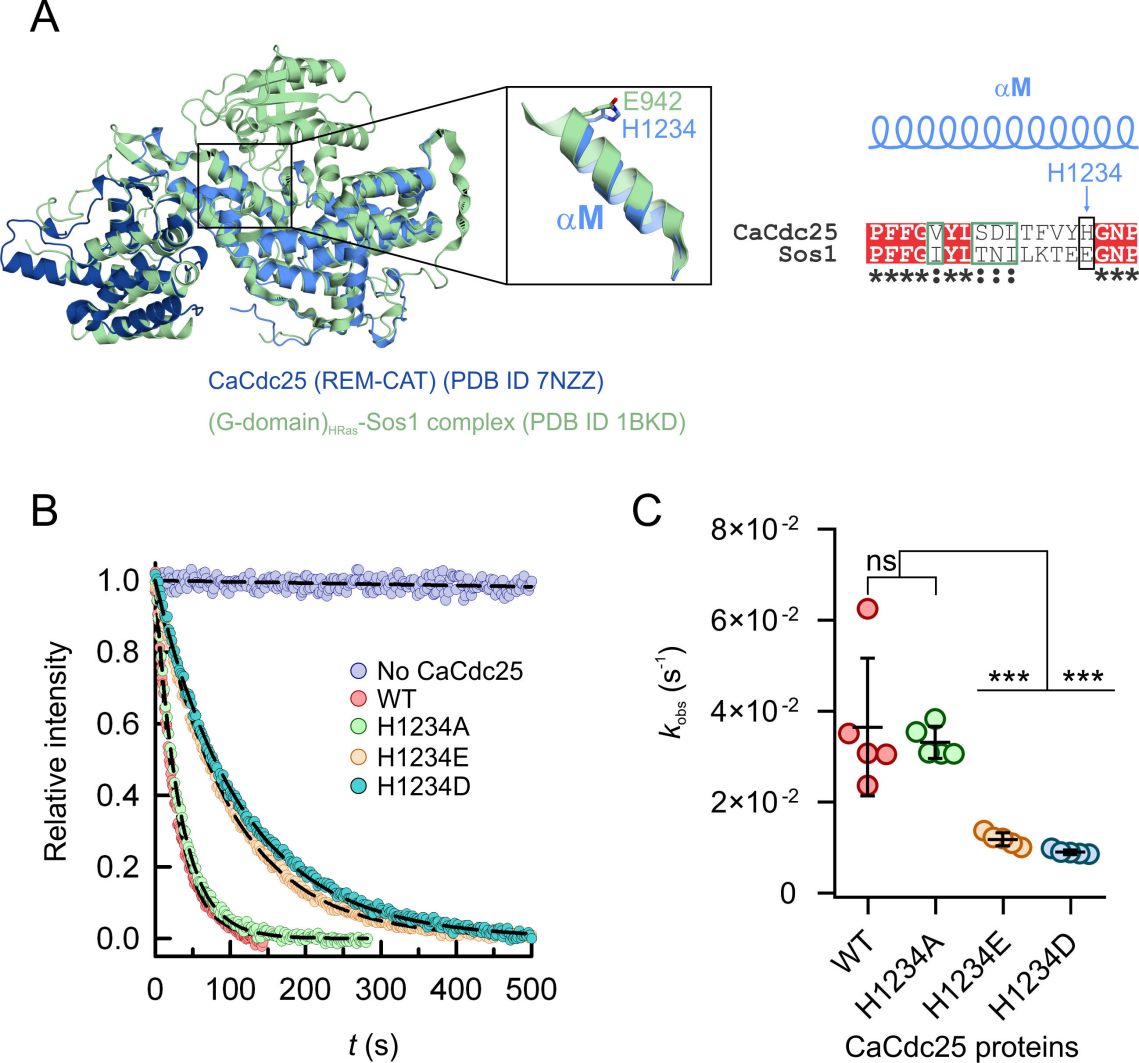
Charge reversal at position 1234 of the unique α-helical hairpin, conserved in the most common human pathogens, reduces the CaCdc25 nucleotide exchange activity. (**A**) Superposition of the structure of the catalytic region of CaCdc25 (PDB code 7NZZ; blue cartoon) with that of the complex of the G-domain of HRas with Sos1 (PDB code 1BKD; green cartoon). The inset shows the Sos1 helix essential for Ras nucleotide exchange, αM in CaCdc25, located in the α-helical hairpin, highlighting that the important E942 of Sos1 occupies a position equivalent to that of H1234 in CaCdc25. The amino acid sequence alignment of the region corresponding to helix αM in *C. albicans* CaCdc25 (UniProtKB entry P43069) with the equivalent region of human Sos1 (UniProtKB entry Q07889) is shown on the right, with strictly conserved positions in inverted type on a red background, similar positions surrounded by a green rectangle, and H1234 and E942 highlighted with a black rectangle. (**B**) Representative nucleotide exchange reactions of CaRas1 G-domain (at 200 nM), isolated or with CaCdc25 (100 nM wild-type CaCdc25 or variants H1234A, H1234E, and H1234D). (**C**) Scatter plot of the *k*_obs_ values determined from fitting a single exponential decay model to the nucleotide exchange experimental data shown in B. Five independent experiments were performed. Statistically significant differences (one-way ANOVA and Dunnett’s multiple comparison test) are indicated by *** (*P ≤* 0.001) and non-significant by ns (*P* ≥ 0.05).

The GDP/GTP exchange reaction in Ras proteins is stimulated through their interaction with the CAT domain of GEFs, a mechanism well conserved from yeast to humans (34, 35). Once the GEF binds the GDP-bound, inactive Ras, the α-helical hairpin of the CAT domain has a crucial role in nucleotide release and consequently in Ras activation. In particular, the side chains of residues from this hairpin modify the chemical environment of the nucleotide binding site, promoting GDP release (36). The subsequent binding of GTP to Ras not only activates it but also disrupts the Ras-GEF complex. In the Sos1-HRas complex, L938 and E942 from Sos1 (the latter also present in the human GEFs RASGRF1, EPAC2, and RASGRP1) are key residues for nucleotide release, interfering with the binding of Mg^2+^ and of the α-phosphate group of the nucleotide, respectively. In order to characterize the CaRas1 nucleotide exchange by CaCdc25, we evaluated the effect of single residue substitutions at position 1234 of the α-helical hairpin, which is structurally equivalent to the important E942 in Sos1 (Fig. 4A), on the activity of CaCdc25. Unexpectedly, the activity observed for the catalytic region of CaCdc25 (*k*_obs_ = (4 ± 2) 10^−2^ s^−1^) was considerably higher (from 5 to 2,000-fold) (Table 2) than those previously reported for other GEFs, under similar experimental conditions (37 - 39), underscoring the efficiency of CaRas1 activation by this GEF. In addition, replacing H1234 by a negatively charged residue resulted in a fourfold reduction of the activity (*k*_obs_ = (1.2 ± 0.1) 10^−2^ s^−1^ for H1234E and *k*_obs_ = (0.90 ± 0.05) 10^−2^ s^−1^ for H1234D), whereas replacement with an uncharged amino acid did not significantly alter the activity (*k*_obs_ = (3.3 ± 0.4) 10^−2^ s^−1^ for H1234A) (Fig. 4B and C). Of note, sequence conservation analysis with ConSurf (40) led to the identification of 150 homologs of CaCdc25 in fungi. Within this group of homologous proteins, a histidine residue is strictly conserved in a position equivalent to that of H1234 in CaCdc25 only in 10 annotations, all of them belonging to the CTG-clade that contains most of the pathogenic *Candida* species (2, 41), while alanine and valine are found in the *Saccharomyces cerevisiae* and *Candida glabrata* homologs, respectively (Fig. S5). The remaining sequences have mostly a negatively charged residue (129 have an aspartate and 3 a glutamate) in the position equivalent to H1234 of the catalytic region of CaCdc25 and belong to non-pathogenic fungi. In summary, H1234 in the α-helical hairpin is a key residue for the GEF activity of CaCdc25 and exclusive to CTG-clade species.

**TABLE 2:** Comparison of the specific activities between different GEFs with Cdc25 homology domains

GEF	Specific activity (*k*)^*a*^ / 10^5^ M^−1^ s^−1^	Ratio *k*_CaCdc25_/*k*_GEF_
CaCdc25	4^*b*^	1
Sos1	0.002^*c*^	2000
RasGRP1	0.02^*c*^	200
RasGRP2	0.05^*c*^	80
RasGRP3	0.01^*c*^	400
PDZ-GEF1	0.8^*c*^	5
PDZ-GEF2	0.8^*c*^	5
Rlf	0.04^*d*^	100
C3G	0.03^*e*^	133

^*a*^ As *k*_obs_/GEF.

^*b*^ This work.

^*c*^ Specific activity values derived from a screening using different GTPases (only values for which GEFs showed to be efficient were taken account). We do not report values for Epac1 and Epac2 since they are activated following binding by cAMP. In the absence of the agonist, Epac is classified as a very poor GEF (38).

^*d*^ Value taken from reference 37.

^*e*^ Value taken from reference 39.

### The CaRas1/CaCdc25 interaction surface is distinct from human homologs

**Fig 5:**
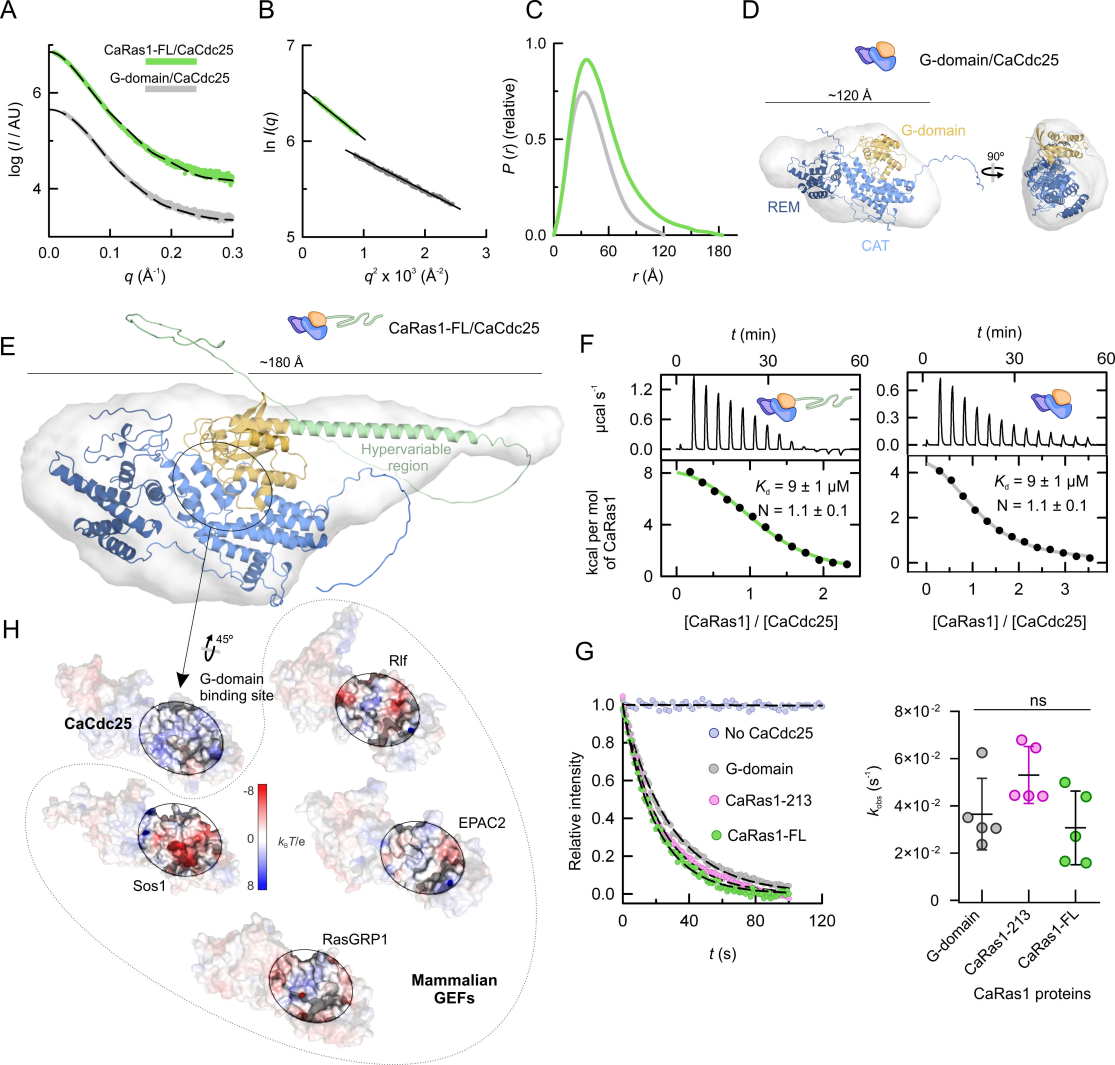
Experimental validation of the AI-based structural models of the CaCdc25/CaRas1 complexes and comparison of the surface of interaction in mammalian GEFs. (**A**) Experimental SAXS profiles extrapolated to infinite dilution of the complexes of the catalytic region of CaCdc25 with CaRas1-FL and CaRas1 G-domain. Curves are offset on the log scale to improve readability. The scattering curves calculated for the corresponding atomic models (shown in D and E) are plotted as black dashed lines. (**B**) Guinier plots of the scattering data shown in A. (**C**) *P*(*r*) functions determined from the scattering data shown in A. (**D and E**) Cartoon representations of the AlphaFold2-predicted models for the complexes G-domain/CaCdc25 and CaRas1-FL/CaCdc25 docked into the corresponding SAXS-derived molecular envelopes shown as gray semi-transparent surfaces. The low-resolution structures are the average of 15 independent bead models. (**F**) Characterization of the interaction of the catalytic region of CaCdc25 with CaRas1. Thermograms and binding isotherms of the binding of the REM-CAT tandem of CaCdc25 to CaRas1-FL (left) and to the G-domain of CaRas1 (right). The data were analyzed fitting a model assuming one binding site in the catalytic region of CaCdc25 for CaRas1-FL (green line) and CaRas1 G-domain (gray line). The presence of the hypervariable region in CaRas1 does not affect the measured affinity, suggesting that it does not impact the recognition of CaCdc25. (**G**) Evaluation of the effect of the hypervariable region on the activity. Representative nucleotide exchange reactions (left). Mant-dGDP loaded CaRas1 G-domain, CaRas1-213 and CaRas1-FL were used at 200 nM in absence and in presence of 100 nM CaCdc25. The *k*_obs_ values for the nucleotide exchange reactions (right) were obtained by fitting a single exponential decay model (dashed lines in the left) to the experimental data. All kinetics were performed in quintuplicate. Lines represent mean ± SD of the replicates. The differences observed for the different constructs were not significant (ns). (**H**) Comparison of the surface electrostatic potential of *C. albicans* CaCdc25 and of mammalian structural homologs. Representation of the molecular surfaces of the CAT and REM domains colored according to their electrostatic potential. Structures of the mammalian homologs Sos1 (PDB entry 3KSY [42]), RasGRP1 (PDB entry 4L9M [43]), EPAC2 (PDB entry 4F7Z [44]), and Rlf (PDB entry 5CM8 [37]) are represented. The G-domain binding site of each GEF for the corresponding GTPase is delimited by a black line.

The catalytic region of CaCdc25 forms complexes detectable by size-exclusion chromatography (SEC) with either CaRas1-FL or its isolated G-domain (see supplemental methods). SAXS analysis (Fig. 5A) of these complexes revealed monodispersity of the samples (Fig. 5B) and 1:1 stoichiometry, as deduced from the estimated masses (Table S1). A *P*(*r*)-derived *D*_max_ of ∼120 Å for the G-domain/REM-CAT complex (Fig. 5C) is in good agreement with its AlphaFold2-derived structural model (Fig. 5D; Fig. S6). The theoretical scattering of this model matches well the experimental profile (Fig. 5A) and it can be nicely fitted in the low-resolution molecular envelope (Fig. 5D).

Both the distance distribution function and the *ab initio* reconstruction of the envelope reveal a more extended shape for the complex with CaRas1-FL than with the G-domain (Fig. 5C and E). Of note, a predicted model for the complex generated with AlphaFold2 could be easily docked in the low-resolution envelope (Fig. 5E; Fig. S6A and B), and the theoretical scattering calculated for this complex matches well the experimental data (Fig. 5A). In this model, part of the hypervariable region is predicted to extend the last helix of the G-domain, as was the case for the isolated CaRas1 protein. However, region 213–290 seems to be more flexible in the complex than in the isolated CaRas1 (for the different conformations, lower predicted lDDT values were obtained [Fig. S6B]). Validation of this apparent increase in flexibility using an EOM analysis revealed instead limited flexibility (Fig. S7A through C), as previously observed for free CaRas1-FL.

Further, the predicted model suggests that the hypervariable region of CaRas1 is not involved in the interaction, being therefore unlikely to interfere in the CaCdc25-modulated nucleotide exchange reaction. Indeed, ITC data confirmed that complex formation was unaffected by the presence of the hypervariable region and revealed that the catalytic region of CaCdc25 interacted with CaRas1-FL or CaRas1 G-domain with µM affinity (*K*_d_ = 9 µM) (Fig. 5F) and 1:1 stoichiometry, as suggested by the SAXS data. Also, varying the length of the hypervariable region did not affect the nucleotide exchange activity of the catalytic region of CaCdc25 (Fig. 5G), with similar *k*_obs_ observed for three different CaRas1 constructs, further suggesting that the hypervariable region has no effect on the nucleotide exchange activity.

The CaRas1/CaCdc25 interaction mode displayed in the two AI-based models is similar to that described for human HRas and Sos1 (36) (Fig. 4A). Although this is a well-conserved mechanism in the Cdc25 family, the predominantly positive electrostatic potential in the interaction surface observed for CaCdc25 (Fig. 5H) clearly differs from the several acidic patches observed in its mammalian structural homologs. The distinctive surface potential in the G-domain binding site and the here identified unique and functionally relevant region in the α-helical hairpin (see above), hint at the catalytic region of CaCdc25 being a potential target for specifically inhibiting Ras1-activation in *Candida albicans* without affecting other human GTPases.

### A CaCdc25-derived peptide inhibits its GEF activity

**Fig 6:**
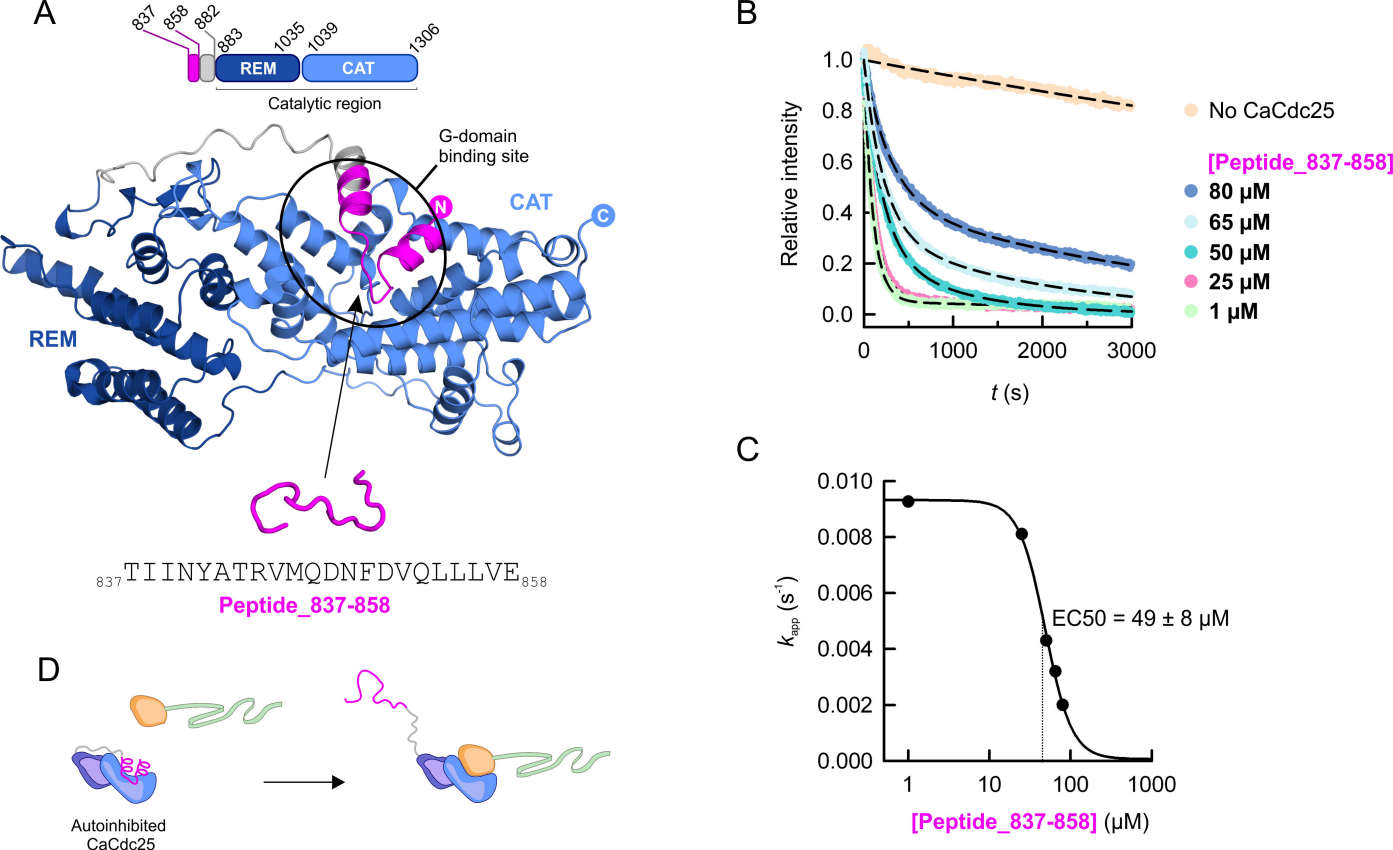
A synthetic peptide reduced the nucleotide exchange activity of CaCdc25 supporting a novel auto-inhibited conformation in the Cdc25 GEFs family. (**A**) Domain organization diagram (top) and cartoon representation (middle) of the AlphaFold2-predicted model for a segment of CaCdc25 comprising the REM (dark blue, residues 883–1305) and CAT domains (light blue, residues 1039–1306), and a hypothetical inhibitory region formed by two α-helices (magenta, residues 837–858) linked to the REM domain by a linker (gray, residues 859–882). The location of the G-domain binding site in CaCdc25 is highlighted. The sequence of the synthetic peptide, tested in the nucleotide exchange assays, is shown at the bottom. (**B**) Nucleotide exchange reactions of CaRas1-mant-dGDP (200 nM) catalyzed by CaCdc25 (50 nM) in the presence of the synthetic peptide 837–858 (1–80 μM concentration range). Dashed lines are the double exponential decay models fitted to obtain *k*_app_. (**C**) Dose-dependent effect of peptide 837–858 on the GEF activity of CaCdc25 (50 nM). The curve (black line) is the fitted Hill model. The half maximal effective concentration (EC50) is shown. (**D**) Schematic representations for the AlphaFold2-predicted structures of the isolated segment 837–1306 of CaCdc25, which represents a hypothetical auto-inhibited conformation, and in complex with CaRas1, where the region 837–858 is displaced from the G-domain binding site of CaCdc25.

The identification of the CaCdc25/CaRas1 binding interface and the distinctive properties of the G-domain binding site of CaCdc25 compared to mammalian GEFs raised the possibility of exploring this site to specifically inhibit the GEF activity of CaCdc25. In the AlphaFold2 model of full-length CaCdc25 (AF-P43069-F1), a region upstream the REM-CAT tandem occupies the G-domain binding site region. To further explore this finding, we generated several AlphaFold2 models for CaCdc25 segment 837–1306, which comprises the hypothetical inhibitory and catalytic regions (Fig. 6A; Fig. S6C and D). In the five models generated, the hypothetical inhibitory region, containing two α-helices (predicted lDDT of ∼60–70), localizes to the CaRas1 binding site. In order to evaluate the putative inhibitory role of this segment, the effect of adding a synthetic peptide comprising residues _837_TIINYATRVMQDNFDVQLLLVE_858_ to the nucleotide exchange activity assays was assessed. A dose-dependent reduction of the activity of CaCdc25 was observed (Fig. 6B), with an estimated EC50 value of 49 ± 8 μM (Fig. 6C). This sequence is only significantly conserved in Cdc25 proteins from species belonging to the *Saccharomycetales* kingdom, predominantly in proteins from the *Candida/Lodderomyces* clade (Fig. S8). In the AlphaFold2-derived models of the CaCdc25 (837–1306)/CaRas1 complex, displacement of segment 837–858 from the G-domain binding site and loss of the helical secondary structure are observed (Fig. 6D, Fig. S6A and B). These observations prompted us to suggest an auto-inhibitory mechanism of CaCdc25 transversal to the homologous fungal GEFs that could represent a unique way to regulate the physiological activity of these molecules. Similarly to the peptide sequence, this auto-inhibitory mechanism has not been found in other mammalian GEFs as far as we know, which opens the avenues for diverse future strategies aiming to specifically stabilize this conformation in *C. albicans*.

## DISCUSSION

The extraordinarily flexible adaptation of *C. albicans* to fluctuations in the host microenvironment is a key aspect of its biology, critical for the commensal to pathogen transition of this opportunistic human pathogen (45). The *C. albicans* Ras/cyclic AMP (cAMP) signal transduction pathway regulates the expression of several virulence traits involved in host recognition, tissue invasion, and evasion of host defense mechanisms. Disrupting the activation of CaRas1, which triggers the signaling pathways that drive the virulence-linked morphological transition from yeast form to a hypha state, is, therefore, a promising approach for the development of antifungal therapies. However, the highly conserved G-domain of CaRas1 (66% amino acid sequence identity with human HRas, with nearly full conservation on the structural elements P-loop, Switch I, Switch II, and NKxD motif [Fig. S4B]), and the striking conservation of Ras activation mechanisms has so far impeded the rational design of molecules targeting the activation of this central environmental sensor in *C. albicans* and in related pathogenic species (11, 46). Indeed, only a short N-terminal extension in the Ras protein of the human fungal pathogen *Aspergillus fumigatus*, which is absent in Ras homologs of higher eukaryotes, has been proposed as potential target (47). Here, we characterize the biochemical and structural features of CaRas1 and its activator CaCdc25 and uncover structural elements specific to *Candida* spp. that could be potentially exploited for the development of novel anti-fungal therapies.

### A structured hypervariable low complexity C-terminal tail in CaRas1

Downstream the highly conserved G-domain of CaRas1 there is a hypervariable region, unusual among Ras-family proteins, which contains low complexity sequence segments, including PolyQ repeats and a Q/N rich stretch, and annotated as unstructured in some databases for intrinsically disordered regions. Cleavage of membrane-associated CaRas1 at this hypervariable region has been proposed as a mechanism restricting CaRas1-mediated activation of the downstream adenylate cyclase (CaCyr1) and, consequently, hyphal growth (13). CaRas1 activity is also affected by different ATP levels via CaCyr1 and the GTPase activating protein CaIra2 (48). In line with previous studies that demonstrated that the hypervariable region of CaRas1 does not play a direct role in CaCyr1 activation (13), our data reveal that it also does not influence the interaction with the GEF catalytic region of CaCdc25 or its nucleotide exchange activity. So far, the functional role of the hypervariable region of CaRas1 remains elusive, but the structural characterization reported here can provide new clues to unveiling the function of this unusual C-terminal domain.

CaRas1 displays an elongated rod-like shape, with the highly conserved and globular G-domain localizing at one end. The remaining portion of the protein is part of the hypervariable region, where structured elements in the form of α-helical and coiled-coil structures contribute to this extended conformation. Up close, the hypervariable region consists of two characteristic segments: one composed of a first polyQ region and a Q/N-rich stretch (residues 178–221) and the other comprising a second Q-rich region (residues 248–267, ∼70% Q). The region connecting these two segments (residues 222–247) has a significant content of disorder-favoring glycine (∼30%) and serine/alanine (∼20%) residues. The first part of the hypervariable region of CaRas1 harbors α-helical elements, as assessed by CD and in agreement with the estimation of several secondary structure predictors. We propose that the terminal helix of the G-domain extends through the first polyQ region until near the end of the Q/N stretch, in line with the susceptibility to proteolysis of the region past N212 in *C. albicans* cells (13). This hypothesis is further supported by the AlphaFold2-predicted structures of full-length CaRas1 (Fig. 2A), where a long helix in the first segment of the hypervariable region was predicted in all generated models and with a high degree of confidence. A similar, although much shorter, helical extension has been described for the human hypervariable region of KRAS4b, which contains a polybasic region formed by a stretch of lysines (49). Further, an increase of helical component and the formation of a coiled-coil structure were observed by CD when the remaining C-terminal was present (residues 213–290). Although some sequence analysis servers (e.g., LOGICOIL [50] [Fig. S7D]) predicted coiled-coil formation, only one of the five AlphaFold2 models displayed a helical structure in this segment of the hypervariable region (Fig. 2A), which interestingly involves the second polyQ stretch. In summary, the combination of experimental studies and AI-based models provides strong evidence of a structured hypervariable region in CaRas1, with a coiled-coil probably formed by the two polyQ stretches.

Interestingly, heterotypic and homotypic interactions involving α-helical and intrinsically disordered regions have been shown to mediate phase separation of septal pore-associated proteins in intracellular compartments required for septal biogenesis and cellular communication (51). PolyQ repeat-containing proteins have been frequently associated with protein complex formation and signaling processes (52, 53) and protein self-assembly (54), with impact on resistance to stress and environmental adaptation in yeast (55 - 57). The structured hypervariable region described here, involving polyQ stretches and having similar amino acid composition from other opportunistic fungal pathogens and which are absent in *S. cerevisiae* and human homologs, offer novel opportunities to investigate the use for the rational design of molecules able to bind to these structured regions that eventually may have an effect in these polyQ associated processes.

### CaCdc25: a hyperactive Ras1 nucleotide exchange factor with pathogen-specific regions

CaRas1 is activated by CaCdc25, triggering the virulence-related MAPK and cAMP signaling pathways. The activated form of CaRas1, CaRas1-GTP, binds to CaCyr1 and induces a cAMP pulse that promotes the yeast-to-hypha transition (58). Whereas, the recognition mode of CaRas1 by the catalytic region of CaCdc25 is similar to that of HRas by Sos1, the three-dimensional structure of the REM-CAT tandem of CaCdc25 revealed poor conservation of the residues located in the CaRas1 interaction surface, in particular in the α-helical hairpin, which is critical for triggering the conformational changes required for GDP release. Therefore, and in contrast to the G-domain of CaRas1, where high conservation across species hinders the structure-based design of pathogen-specific targeting molecules, the observed divergences in CaCdc25 make it a potential target to specifically inhibit CaRas1 activation.

Interestingly, the observed stimulation of CaRas1 nucleotide exchange by CaCdc25 was unusually high, suggesting that this GEF is optimally poised for CaRas1 activation. In fact, the nucleotide exchange activity of CaCdc25 is 5 to 2,000-fold higher than that of characterized human GEFs (37 - 39) (Table 2). This high level of activity of CaCdc25 could result from the stabilization of the α-helical hairpin in an active conformation, as inferred from its crystallographic structure, or from the unique chemical environment of the hairpin, which could influence nucleotide release and therefore CaRas1 activation. Indeed, the nature of the residue at position 1234 seems be critical for the activity of CaCdc25, since the introduction of charge-reversing substitutions at this position (as the variants H1234D and H1234E) originated less active variants. It is also noteworthy that both H1234 and a small region of the hairpin are strictly and uniquely conserved in the fungal CTG-clade (Fig. S5), which groups the most common human fungal pathogens, suggesting a possible correlation between GEF activity and pathogenicity. In addition, the distribution of electrostatic potential in the CaCdc25/CaRas1 interaction surface is clearly distinct from that observed in homologous mammalian GEFs. Numerous regulatory mechanisms can be found in GEFs, including protein-protein or protein-lipid interactions, binding of second messengers, and posttranslational modifications (59). These interactions and/or modifications promote the release of characteristic auto-inhibitory regions needed to prevent uncontrolled signaling. In this particular case, we propose the CaCdc25 segment _837_TIINYATRVMQDNFDVQLLLVE_858_, only present in fungal GEFs, as an auto-inhibitory region. Conformational changes required to displace this region from the CaCdc25-CaRas1 interface are presumably dynamic processes, in accordance with the relatively high EC50 value determined for the peptide, and the underlying mechanism of auto-inhibition must be further studied.

Altogether, the fungi-related features of the sole Ras1-specific GEF identified so far (12) highlight the relevance of CaCdc25 for designing molecules capable of modulating specifically CaRas1 nucleotide exchange and, consequently, the cAMP and MAPK signaling pathways, which regulate critical virulence factors in *C. albicans*. In particular, the identification of a short peptide that effectively inhibits the activity of this GEF, constitutes a valuable starting point for the design and development of specific inhibitors with potential antifungal properties based on this privileged scaffold.

## MATERIALS AND METHODS

Methods are summarized below and described in detail in Text S1 in the supplemental material.

### Protein expression and purification

CaRas1-FL, CaRas1-213, G-domain, and the catalytic region of CaCdc25 and its variants H1234A, H1234D, and H1234E were expressed in *Escherichia coli* BL21 (DE3) or *E. coli* Rosetta (DE3) after induction with IPTG at 37°C or 20°C. Proteins were purified by immobilized-metal affinity chromatography and size-exclusion chromatography.

### Structure determination

Diffraction data were collected at beamline BL13-XALOC of ALBA synchrotron. X-ray diffraction data collection and processing statistics are summarized in Table 1. A low resolution diffraction data set was collected from two isomorphous crystals of the catalytic region of CaCdc25 (data set A), and used for initial structure solution. A partial model derived from data set A was used as template for molecular replacement in a higher resolution data set (data set B), which allowed building and refining the final model of CaCdc25. Refinement statistics are summarized in Table 1.

### ITC and CD measurements

Recombinant CaCdc25 (REM-CAT tandem) was titrated with CaRas1 proteins CaRas1-FL and G-domain using a MicroCal VP-ITC system (Northampton, MA, USA). Data were visualized and analyzed with Origin 7 software using a single binding site model. CD spectra of CaRas1 proteins were recorded using a Jasco J-815 CD spectrometer in a 0.1 cm quartz cuvette for far-UV CD spectroscopy.

### *In vitro* nucleotide exchange activity assay

Guanine nucleotide exchange activity of CaRas1 proteins in absence and in presence of wild-type CaCdc25 and variants was measured by following changes in the fluorescence of the GDP derivative Mant-dGDP (Jena Bioscience GmbH) (31, 60) on a Fluoromax-4 spectrofluorometer (Horiba-Jobin Yvon).

A peptide corresponding to the region 837–858 (TIINYATRVMQDNFDVQLLLVE) of CaCdc25 and two control peptides with scrambled sequence and similar amino acid composition as peptide 837–858 were custom synthesized (Genosphere Biotechnologies, France). The effect of these synthetic peptides on nucleotide exchange assays was assessed.

### SAXS measurements

SAXS data were measured at the P12 beamline of the EMBL at the Deutsches Elektronen-Synchrotron (DESY; Hamburg, Germany). Data were processed and analyzed with the ATSAS 3.0 package (61).

## Data Availability

The X-ray diffraction images (https://doi.org/10.15785/SBGRID/860 and https://doi.org/10.15785/SBGRID/861 for data set A and https://doi.org/10.15785/SBGRID/859 for data set B) were deposited with the Structural Biology Data Grid (62). Coordinates and structure factors were deposited at the Protein Data Bank (PDB) under accession number 7NZZ. SAXS data were deposited at the Small Angle Scattering Biological Data Bank (SASBDB) (63) under codes SASDM55, SASDM65, SASDM75, SASDM85, and SASDM95. Other data are available from the corresponding authors upon reasonable request.

## SUPPLEMENTAL MATERIAL

Fig. S1 mbio.00638-23-s0001.pdf Domain organization of fungal and human Ras proteins.

Fig. S2 mbio.00638-23-s0002.pdf Structure of the CaRas1 G-domain.

Fig. S3 mbio.00638-23-s0003.pdf Overall view of the structures of the two CaCdc25 monomers in the crystallographic asymmetric unit and comparison with other GEF homologues.

Fig. S4 mbio.00638-23-s0004.pdf Amino acid sequence alignments of the CAT domain of CaCdc25 and of the G-domain of CaRas1 with human homologues.

Fig. S5 mbio.00638-23-s0005.pdf Helix α**M** is conserved in common pathogenic fungi.

Fig. S6 mbio.00638-23-s0006.pdf CaCdc25/CaRas1 complexes and CaCdc25 models generated by AlphaFold2.

Fig. S7 mbio.00638-23-s0007.pdf Flexibility analysis of the segment 213-290 in the hypervariable region of CaRas1 when it is complexed with CaCdc25 and prediction of a coiled-coil in CaRas1.

Fig. S8 mbio.00638-23-s0008.pdf Full or partial conservation of the hypothetical auto-inhibitory region of CaCdc25 in species belonging to the *Saccharomycetales* kingdom.

Supplemental methods mbio.00638-23-s0009.docx Text with detailed methods.

Table S1 mbio.00638-23-s0010.docx Small angle X-ray scattering results for CaCdc25 (tandem REM-CAT), CaRas1 constructs and CaRas1/CaCdc25 complexes.

ASM does not own the copyrights to Supplemental Material that may be linked to, or accessed through, an article. The authors have granted ASM a non-exclusive, world-wide license to publish the Supplemental Material files. Please contact the corresponding author directly for reuse.
