# Supplementary material for: Pathogen-specific structural features of Candida albicans Ras1 activation complex: uncovering new antifungal drug targets
Source: mBio. 2023 Aug 1;14(4):e00638-23. doi: 10.1128/mbio.00638-23 (PMC10470544; doi:10.1128/mbio.00638-23)
Supplement: Fig. S1 — Domain organization of fungal and human Ras proteins. [file mbio.00638-23-s0001.pdf]

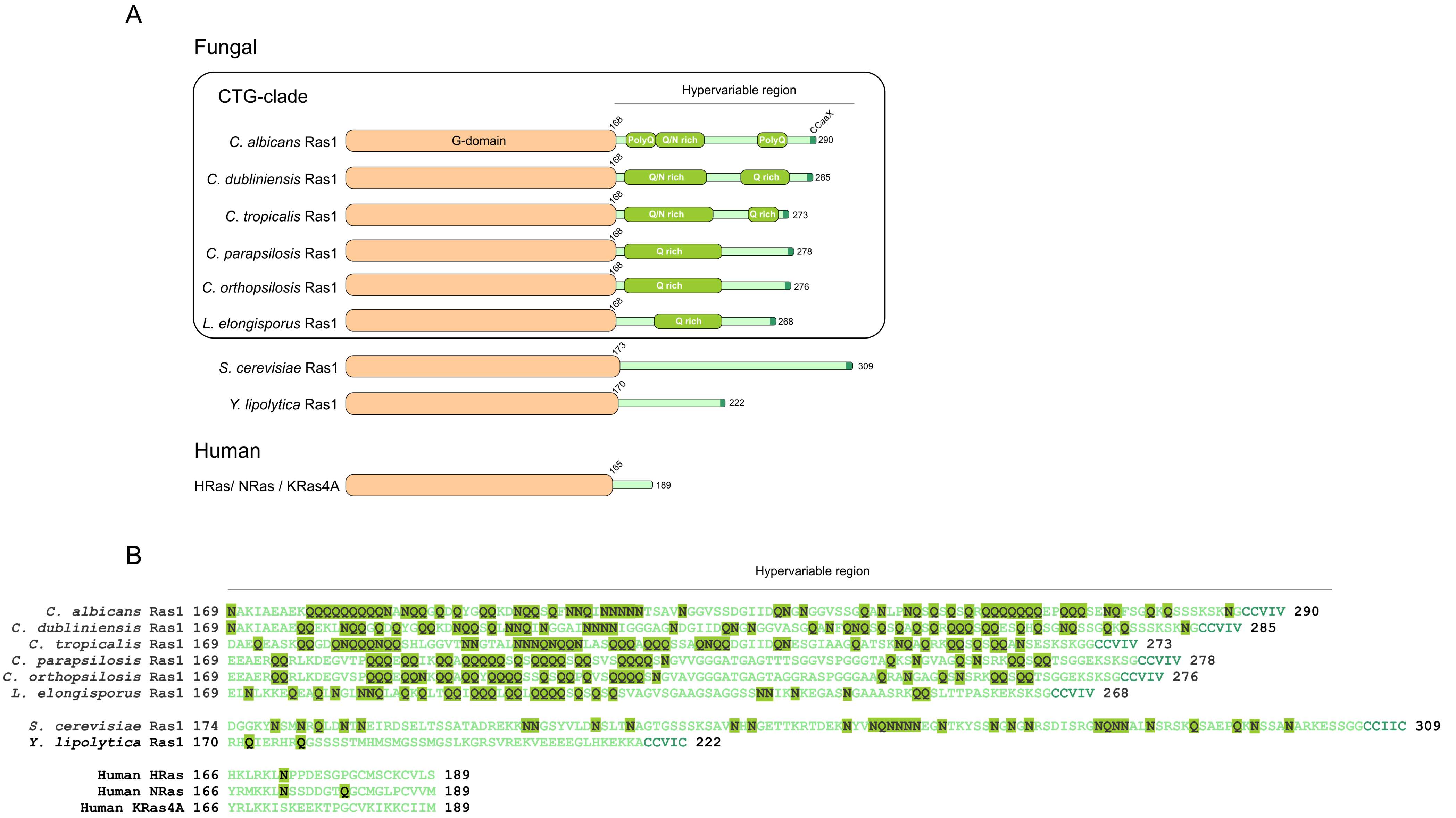

Fig. S1. Domain organization of fungal and human Ras proteins. A) A main difference between fungal and human Ras proteins resides on the length of the hypervariable region, much longer in fungi. The presence of polyQ and Q/N rich regions at the hypervariable region in members of the CTG-clade makes Ras1 unique within the Ras family proteins. *C. albicans* Ras1 (UniProtKB entry P0CY32), *C. dubliniensis* Ras1 (*Candida* Genome Database (CGD) entry Cd36_24270, *C. tropicalis* Ras1 (CGD entry CTRG_02064), *C. parapsilosis* Ras1 (CGD entry CPAR2_407360), *C. orthopsilosis* Ras1 (CGD entry CORT_0C06700), *L. elongisporus* Ras1 (CGD entry LELG_02372), *S. cerevisiae* Ras1 (UniProtKB entry P01119) and *Y. lipolitica* Ras1 (GenBank entry KAG5356685.1), and HRas (UniProtKB entry P01112), NRas (UniProtKB entry P01111) and KRas4A (UniProtKB entry P01116-1) were selected as representative fungal and human Ras proteins, respectively. B) Primary sequences of the hypervariable regions of the proteins listed in A), where Q and N residues are highlighted by a dark green square.
