# Supplementary material for: Pathogen-specific structural features of Candida albicans Ras1 activation complex: uncovering new antifungal drug targets
Source: mBio. 2023 Aug 1;14(4):e00638-23. doi: 10.1128/mbio.00638-23 (PMC10470544; doi:10.1128/mbio.00638-23)
Supplement: Fig. S2 — Structure of the CaRas1 G-domain. [file mbio.00638-23-s0002.pdf]

A

Model	Template (organism)	PDB entry	FFAS-score	Seq. identity, %	Chi²
1	RRas2 (human)	2ERY	−92.5	62	2.5
2	Rap1b (human)	3X1Z	−91.2	61	2.6
3	RalA (human)	6P0I	−92.1	51	2.9
4	Rap1a (human)	1GUA	−91.2	62	3.1
5	Ras (Choanoflagellata)	5WDR	−91.9	72	3.3
6	HRas (human)	6NTC	−91.7	66	3.4
7	HRas (human)	5VBE	−91.6	66	3.5
8	Rap2A (human)	1KAO	−93.1	52	4.0
9	Rheb (human)	6BSX	−93.0	40	7.3
10	Rheb (Mus musculus)	4O25	−92.7	40	7.9
11	KRas G12C (human)	4L8G	−91.3	65	8.7
12	NRas (Homo sapiens)	3CON	−90.1	68	9.6

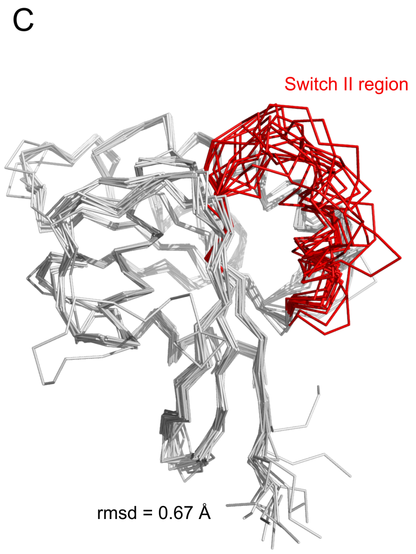

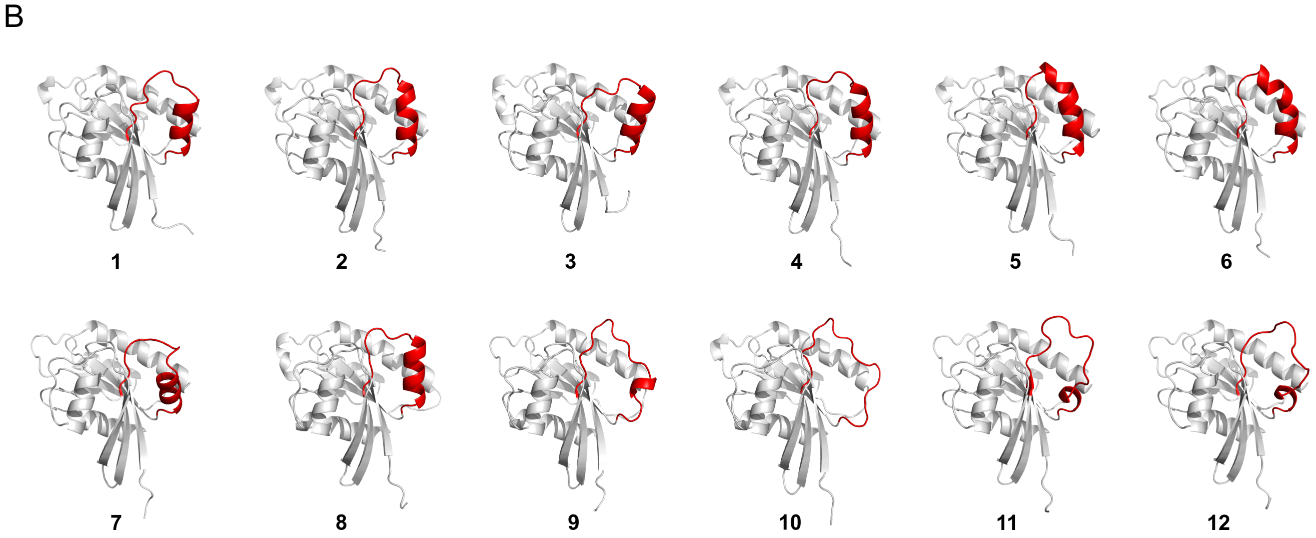

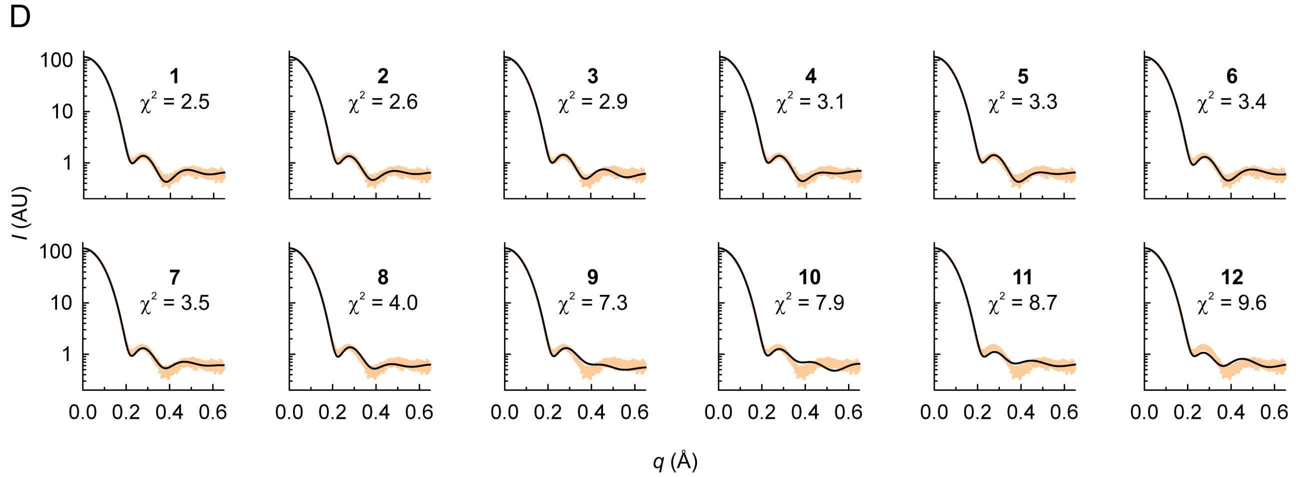

Fig. S2. Structure of the CaRas1 G-domain. A) Models for the *C. albicans* CaRas1 G-domain using as templates twelve structural homologues (small GTPase proteins with 40-66% amino acid sequence identity) identified with the Fold and Function Assignment System (FFAS03) (L. Jaroszewski, Z. Li, X. -H. Cai, C. Weber, and A. Godzik, Nucleic Acids Res 39:W38-44, 2011, https://doi.org/10.1093/nar/gkr441). The models are sorted according to their fit to the SAXS experimental data (as judged by the value of χ^2). Despite the low FFAS-score for all models (scores below −9.5 correspond to high-confidence predictions with less than 3% false positives) and the high degree of amino acid sequence conservation, there are notable differences in the SAXS χ^2 values, which represent the discrepancy between the theoretical and experimental SAXS curves (see panel D). B) Cartoon representation of the models from panel A. Structural differences are mostly located on the switch II region (colored red). C) Structural superposition of the 12 models (represented as Cα traces), highlighting the variations in the switch II region (colored as in panel B). D) Experimental SAXS scattering curve for the CaRas1 G-domain (orange dots) and theoretical curves estimated from the 12 different models (black line). Models with a switch II region displaying α-helical secondary structure (1-8) originate theoretical scattering curves that better fit the experimental data.
