# Supplementary material for: Pathogen-specific structural features of Candida albicans Ras1 activation complex: uncovering new antifungal drug targets
Source: mBio. 2023 Aug 1;14(4):e00638-23. doi: 10.1128/mbio.00638-23 (PMC10470544; doi:10.1128/mbio.00638-23)
Supplement: Fig. S3 — Overall view of the structures of the two CaCdc25 monomers in the crystallographic asymmetric unit and comparison with other GEF homologues. [file mbio.00638-23-s0003.pdf]

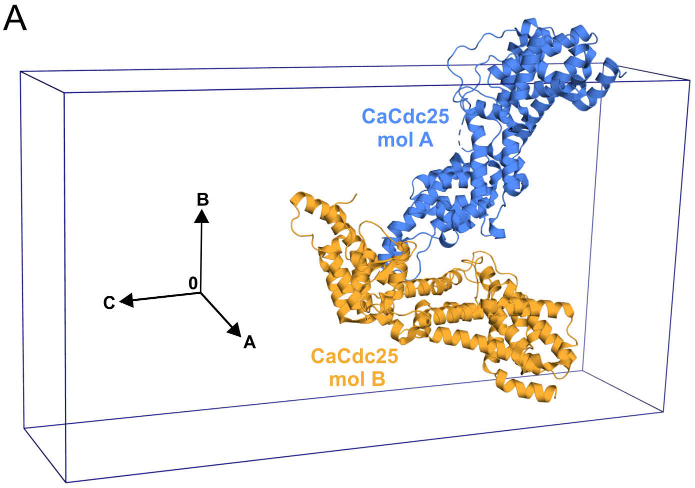

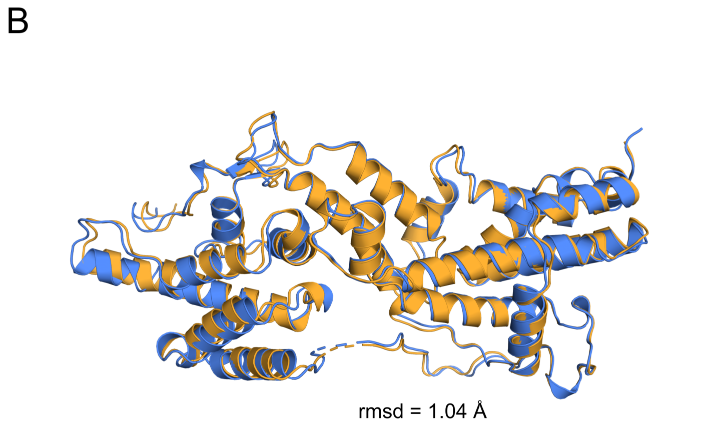

C

PDB entry	Protein	*Z*-score	% seq. identity
5CM8	Rlf (GEF for Ral)	31.6	17
4L9M	RasGRP1 (GEF for Ras)	27.3	18
4F7Z	EPAC2 (GEF for Rap1b)	26.2	22
3KSY	Sos1 (GEF for Ras)	23.1	25

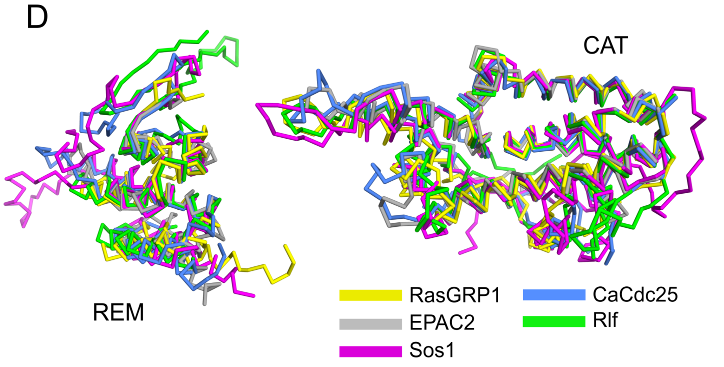

Fig. S3. Overall view of the structures of the two CaCdc25 monomers in the crystallographic asymmetric unit and comparison with other GEF homologues. A) Cartoon representation of the two molecules (blue: molecule A; orange: molecule B) found in the asymmetric unit (AU) of the catalytic region of CaCdc25. B) Structural superposition of the two CaCdc25 monomers found in the crystallographic AU (colored as in panel A). Slight differences were found in the relative orientation or the CAT and REM domains in the two molecules, probably due to the distinct intermolecular contacts in the crystal packing. C) Structural homologues of CaCdc25 identified with the DALI server (L. Holm, Protein Sci 29:128-140, 2020, https://doi.org/10.1002/pro.3749) using molecule A as query structure. There were only four hits with Z-score > 16, all displaying surprisingly low amino acid sequence conservation (≤ 25% sequence identity), corresponding to PDB entries 5CM8 (M. Popovic, A. Schouten, M. Rensen-de Leeuw, and H. Rehmann, J Struct Biol 193:106-114, 2016, https://doi.org/10.1016/j.jsb.2015.12.006), 4L9M (J. S. Iwig, Y. Vercoulen, R. Das, T. Barros, A. Limnander,Y. Che, et al., eLife 2:e00813, 2013, https://doi.org/10.7554/eLife.00813), 4F7Z (M. A. White, S. Li, T. Tsalkova, F. C. Mei, T. Liu, V. L. Woods Jr, and X. Cheng., PLoS One 7:e49932, 2012, https://doi.org/10.1371/journal.pone.0049932) and 3KSY (J. Gureasko, O. Kuchment, D. L. Makino, H. Sondermann, D. Bar-Sagi, and J. Kuriyan, Proc Natl Acad Sci USA 107:3430-3435, 2010, https://doi.org/10.1073/pnas.0913915107). D) Superposition (Cα traces) of the individual structures of the REM domain (left) and CAT domain (right) of CaCdc25 and the four structural homologues listed in panel C.
