# Supplementary material for: Pathogen-specific structural features of Candida albicans Ras1 activation complex: uncovering new antifungal drug targets
Source: mBio. 2023 Aug 1;14(4):e00638-23. doi: 10.1128/mbio.00638-23 (PMC10470544; doi:10.1128/mbio.00638-23)
Supplement: Fig. S4 — Amino acid sequence alignments of the CAT domain of CaCdc25 and of the G-domain of CaRas1 with human homologues. [file mbio.00638-23-s0004.pdf]

A

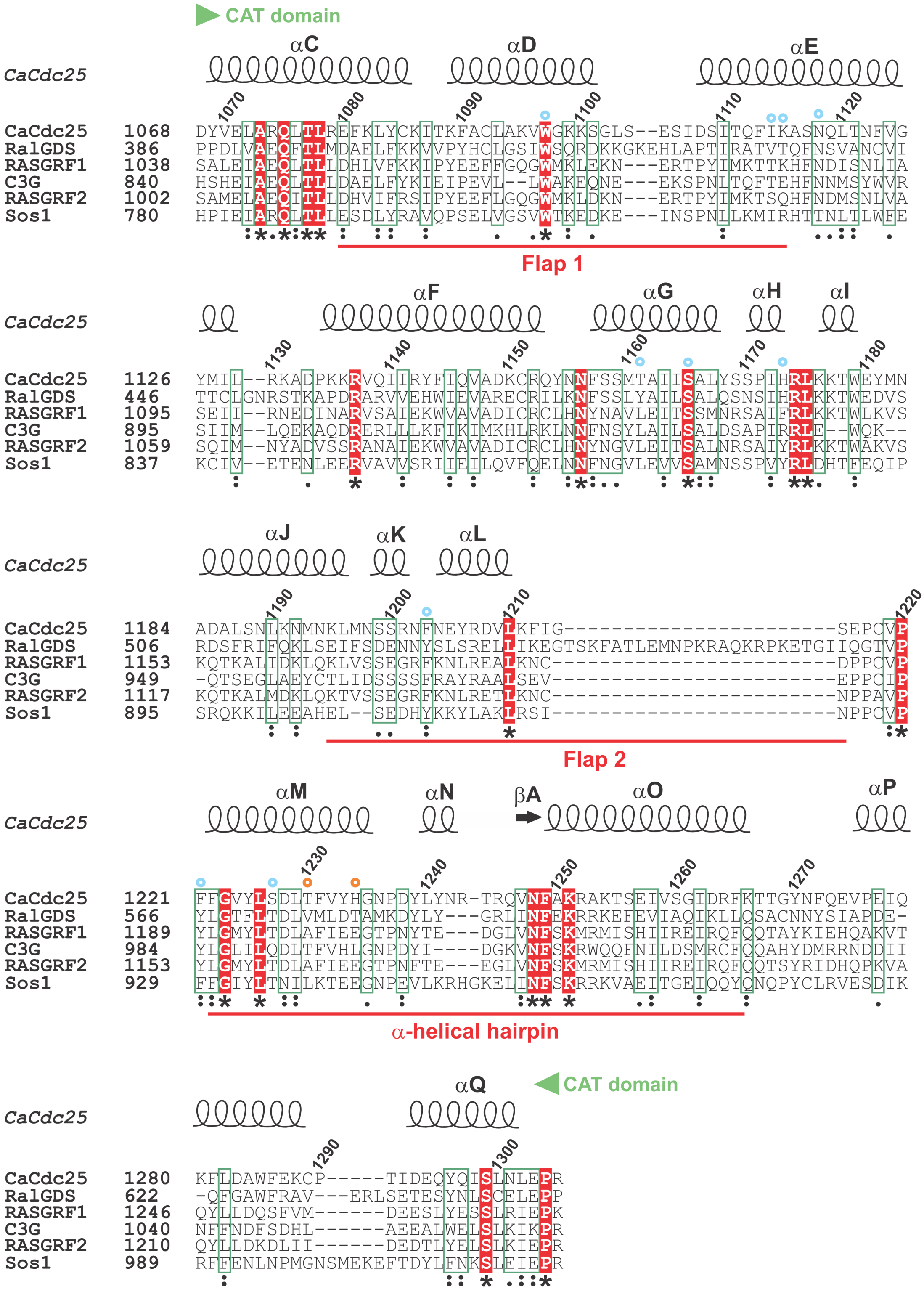
CAT domain
CaCdc25
αC
αD
αE
RalGDS
RASGRF1
C3G
RASGRF2
Sos1
Flap 1
αF
αG
αH
αI
αJ
αK
αL
Flap 2
αM
αN
βA
αO
αP
α-helical hairpin
αQ
CAT domain

B

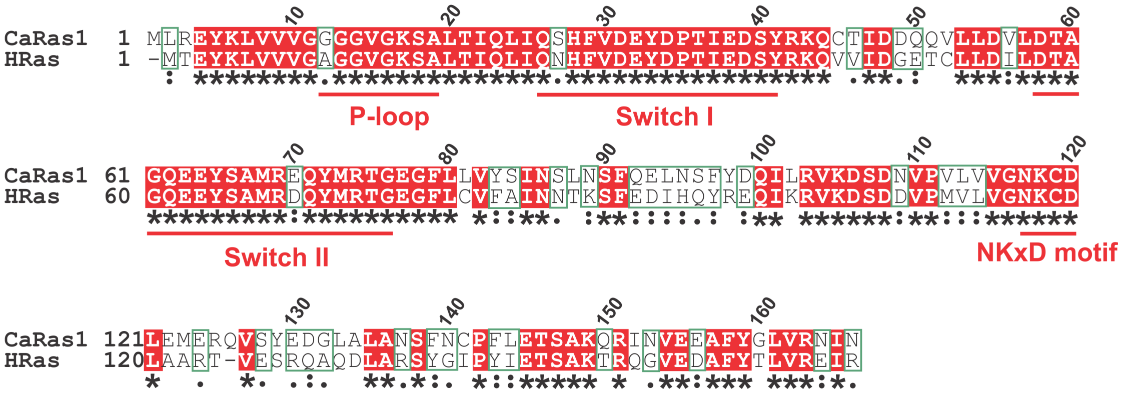
CaRas1
HRas
P-loop
Switch I
Switch II
NKxD motif

Fig. S4. Amino acid sequence alignments of the CAT domain of CaCdc25 and of the G-domain of CaRas1 with human homologues. A) The amino acid sequence of the CAT domain of *Candida albicans* CaCdc25 (UniProtKB entry P43069) was aligned with those of human homologues RalGDS (UniProtKB entry Q12967), RASGRF1 (UniProtKB entry Q13972), C3G (UniProtKB entry Q13905), RASGRF2 (UniProtKB entry O14827) and Sos1 (UniProtKB entry Q07889). Strictly conserved alignment positions are shown in inverted type on a red background. Secondary structure elements for the CAT domain of CaCdc25 are represented above the alignment. The flap 1 and flap 2 regions, as well as the α-helical hairpin are labeled in red. Blue circles indicate Sos1 residues that directly contact HRas in the crystallographic structure of the complex (PDB entry 1BKD; P. A. Boriack-Sjodin, S. M. Margarit, D. Bar-Sagi, and J. Kuriyan, Nature 394:337-343, 1998, https://doi.org/10.1038/28548), and orange circles denote residues from the nucleotide binding site. B) The amino acid sequence of the G-domain of *Candida albicans* CaRas1 (UniProtKB entry P0CY32) was aligned with that of human HRas (UniProtKB entry P01112). Strictly conserved alignment positions are shown in inverted type on a red background. The P-loop, Switches I and II, and the NKxD motif are labeled in red.
