# Supplementary material for: Pathogen-specific structural features of Candida albicans Ras1 activation complex: uncovering new antifungal drug targets
Source: mBio. 2023 Aug 1;14(4):e00638-23. doi: 10.1128/mbio.00638-23 (PMC10470544; doi:10.1128/mbio.00638-23)
Supplement: Fig. S5 — Helix αM is conserved in common pathogenic fungi. [file mbio.00638-23-s0005.pdf]

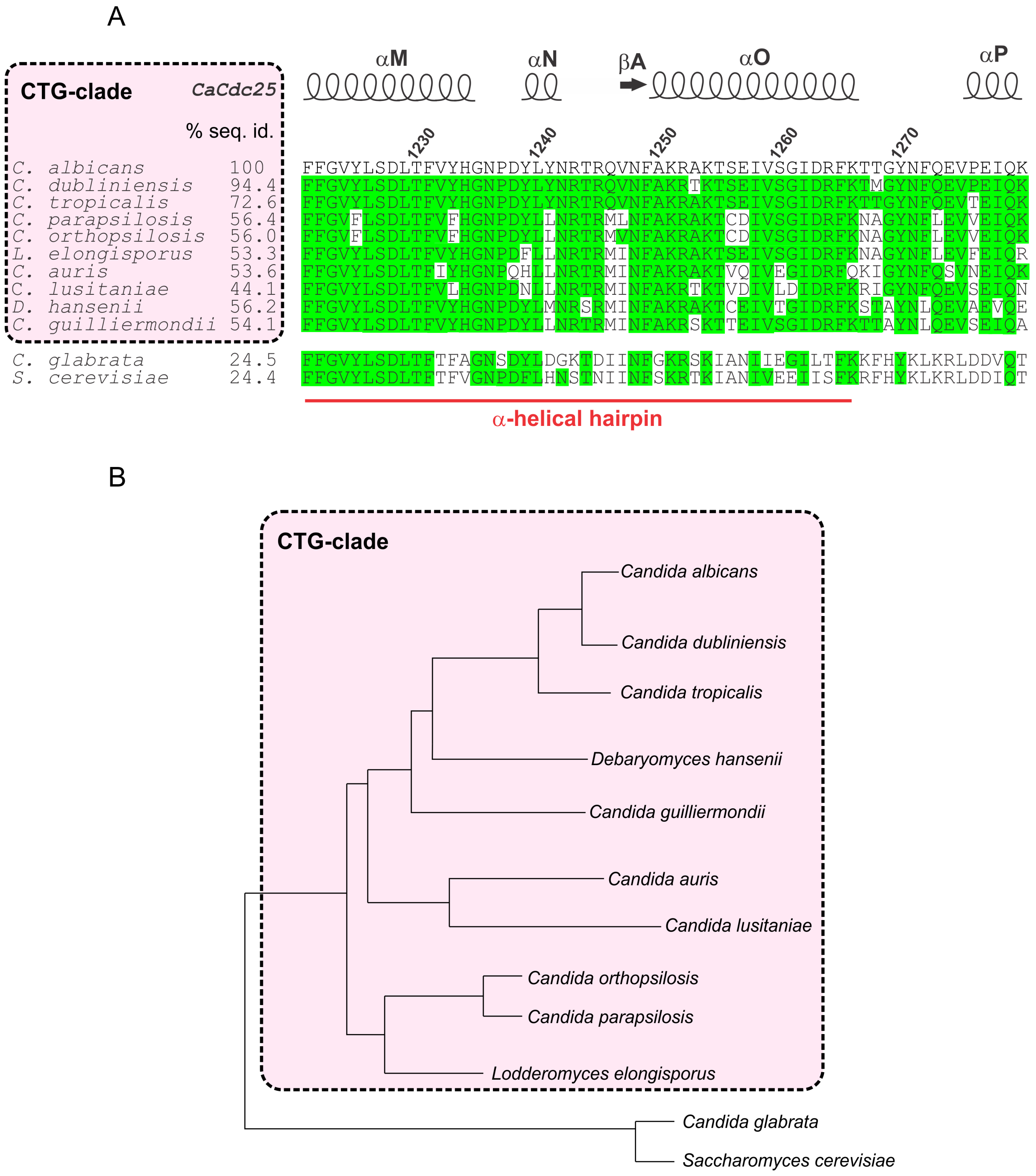

Fig. S5. Helix αM is conserved in common pathogenic fungi. A) Amino acid sequence alignment highlighting the exclusive conservation of helix αM in only ten identified CaCdc25 homologues from other common human pathogenic fungi. B) Phylogenetic tree of CaCdc25 homologues obtained from the *Candida* Genome Database (http://www.candidagenome.org/) and built with SEMPHY (M. Ninio, E. Privman, T. Pupko, and N. Friedman, Bioinformatics 23:e136-141, 2007, https://doi.org/10.1093/bioinformatics/btl304).
