# Supplementary material for: Pathogen-specific structural features of Candida albicans Ras1 activation complex: uncovering new antifungal drug targets
Source: mBio. 2023 Aug 1;14(4):e00638-23. doi: 10.1128/mbio.00638-23 (PMC10470544; doi:10.1128/mbio.00638-23)
Supplement: Fig. S6 — CaCdc25/CaRas1 complexes and CaCdc25 models generated by AlphaFold2. [file mbio.00638-23-s0006.pdf]

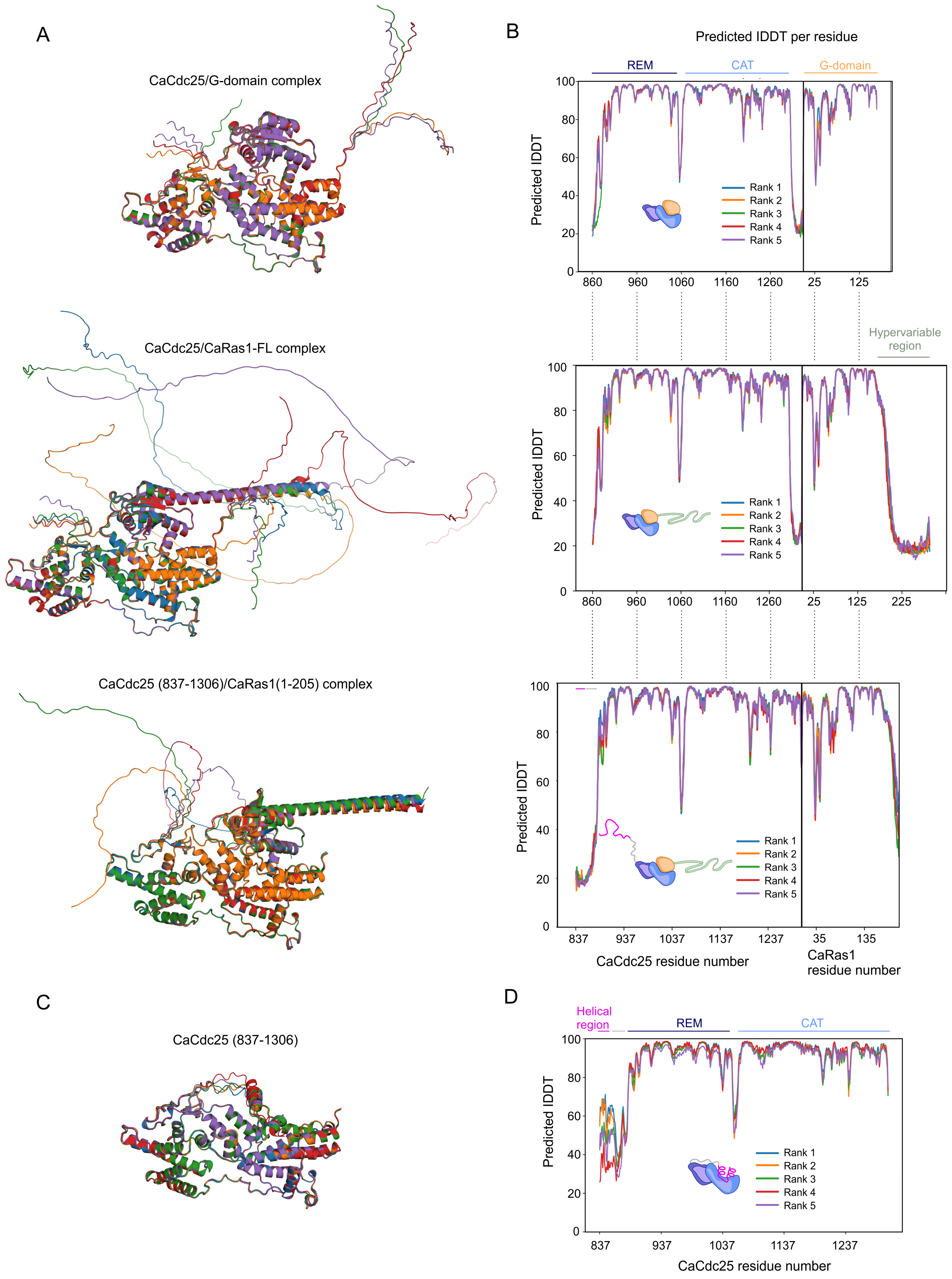

A
CaCdc25/G-domain complex
CaCdc25/CaRas1-FL complex
CaCdc25 (837-1306)/CaRas1(1-205) complex
B
Predicted IDDT per residue
REM
CAT
G-domain
Hypervariable region
Predicted IDDT
Rank 1
Rank 2
Rank 3
Rank 4
Rank 5
CaCdc25 residue number
CaRas1 residue number
C
CaCdc25 (837-1306)
D
Helical region
REM
CAT
Predicted IDDT
Rank 1
Rank 2
Rank 3
Rank 4
Rank 5
CaCdc25 residue number

Fig. S6. CaCdc25/CaRas1 complexes and CaCdc25 models generated by AlphaFold2. A) Ribbon representations for the five predicted structures of the catalytic region of CaCdc25 (REM-CAT domains) in complex with the G-domain of CaRas1 (upper panel) and CaRas1-FL (middle panel), and of the fragment 837-1306 of CaCdc25, which includes a hypothetical auto-inhibitory sequence (837-858, see below), in complex with CaRas1-FL (lower panel; for clarity only the 1-205 region is shown). The five models for each complex were superposed and colored on the basis of their ranking in the predicted per-residue local Distance Difference Test (lDDT) plot showed in B. The interaction mode of the tandem REM-CAT of CaCdc25 with the G-domain of CaRas1 was identical for the fifteen different predictions. B) Predicted lDDT for the different complexes shown in A). C) The five predicted structures for the region comprising residues 837-1306 of CaCdc25 showing a hypothetical auto-inhibited conformation. D) lDDT of the five predicted structures shown in C). Two predicted models present good confidence values (lDDT > 60) for a helical region, which is occupying the G-domain binding site. For clarity, the schematic representations shown in Figs. 5D, 5E and 6D are also represented in B) and D).
