# Supplementary material for: Pathogen-specific structural features of Candida albicans Ras1 activation complex: uncovering new antifungal drug targets
Source: mBio. 2023 Aug 1;14(4):e00638-23. doi: 10.1128/mbio.00638-23 (PMC10470544; doi:10.1128/mbio.00638-23)
Supplement: Fig. S7 — Flexibility analysis of the segment 213-290 in the hypervariable region of CaRas1 when it is complexed with CaCdc25 and prediction of a coiled-coil in CaRas1. [file mbio.00638-23-s0007.pdf]

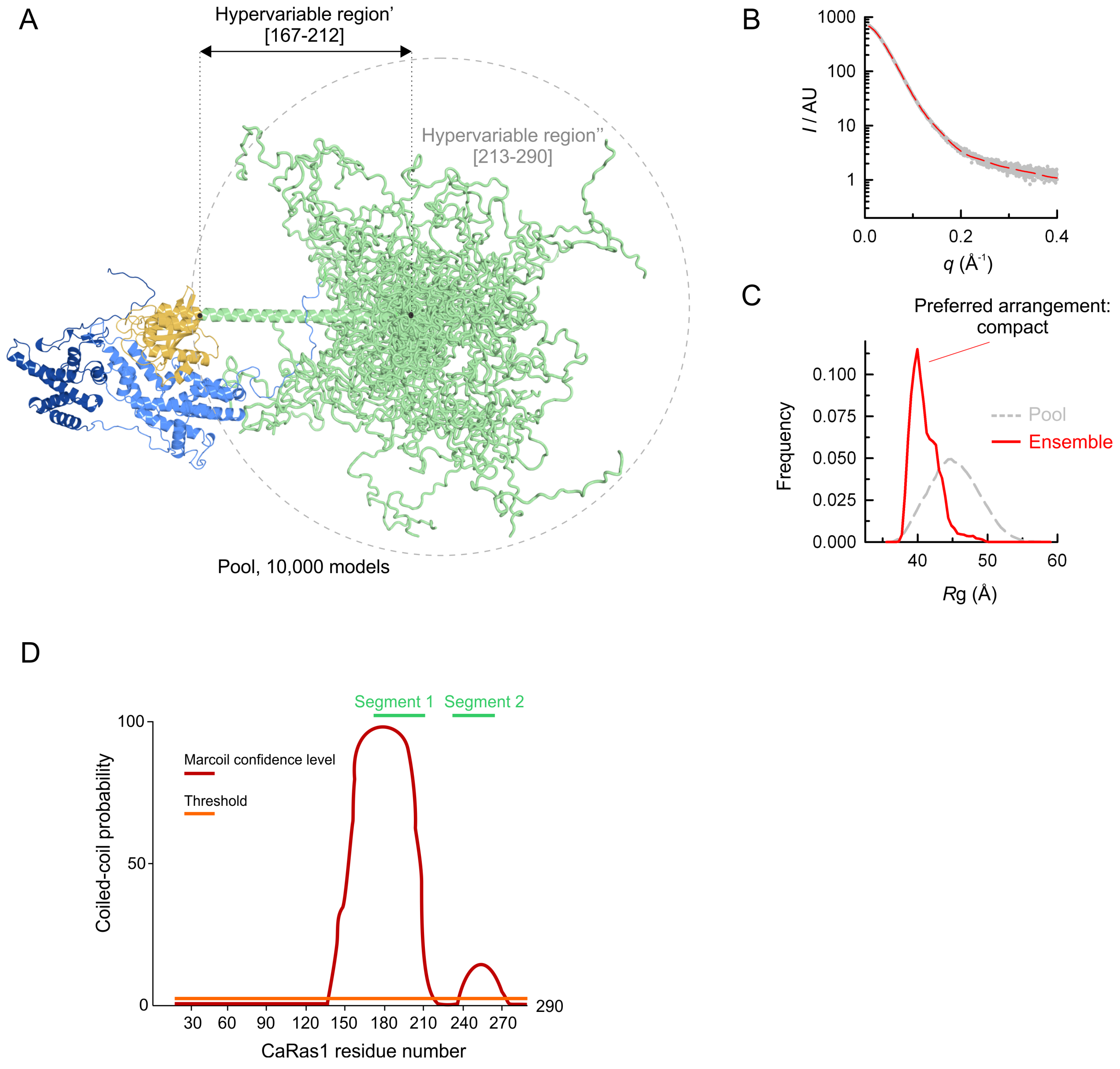

Fig. S7. Flexibility analysis of the segment 213-290 in the hypervariable region of CaRas1 when it is complexed with CaCdc25 and prediction of a coiled-coil in CaRas1. A) Representation of the generated pool to evaluate the flexibility of the CaRas1 region 213-290 (colored pale green) in complex with CaCdc25 by EOM. The pool consisted of 10,000 models, in which the catalytic region of Cdc25 (REM and CAT domains colored dark and light blue, respectively) and the G-domain (colored orange) and the helical part 167-212 of the hypervariable region (colored pale green) of CaRas1 were fixed, whereas the part of the hypervariable region, that comprises the residues 213-290, was considered fully flexible (for clarity only 50 models are represented). B) SAXS experimental data of the full-length CaRas1 in complex with CaCdc25 (represented as gray dots) and the scattering profile calculated for the selected EOM ensemble (represented as a red dashed line), which fits to the data with a χ^2 of 2.038. C) EOM analysis of the flexibility of the 213-290 region (hypervariable region''). Frequency of radius of gyration (*R*g) distributions in a pool of 10,000 models (gray dashed line) with random orientations of the hypervariable region'', and in the selected ensemble that fits the SAXS data of CaRas1-FL that is shown in panel B (red line). Default parameters were employed using native-like models, allowing constant subtraction (0.263) and curve repetition (both the minimum number of curves per ensemble and the number of obtained representative structures, was five). The values for *R*flex(random)/*R*sigma of ~ 70.01% (~ 86.77%) / 0.55 indicate a limited flexibility. D) Two segments within the hypervariable region of CaRas1 are predicted to form coiled-coil structures by LOGICOIL (T. L. Vincent, P. J. Green, and D. N. Woolfson, Bioinformatics 29:69-76, 2013, https://doi.org/10.1093/bioinformatics/bts648).
