# Supplementary material for: Pathogen-specific structural features of Candida albicans Ras1 activation complex: uncovering new antifungal drug targets
Source: mBio. 2023 Aug 1;14(4):e00638-23. doi: 10.1128/mbio.00638-23 (PMC10470544; doi:10.1128/mbio.00638-23)
Supplement: Fig. S8 — Full or partial conservation of the hypothetical auto-inhibitory region of CaCdc25 in species belonging to the Saccharomycetales kingdom. [file mbio.00638-23-s0008.pdf]

```
                                837                   858
                                  .         .
Candida albicans                TIINYATRVMQDNFDVQLLLVE
Candida viswanathii             TIINYATRVMQDNFDVQLLLVE
Candida maltosa                 TIINYATRVMQDNFDVQLLLVE
Candida africana                TIINYATRVMQDNFDVQLLLVE
Candida dubliniensis            TIINYATRVMQDNFDVQLLLVE
Candida tropicalis              TIINYATRVMQDNFDVQLLIVE
Lodderomyces elongisporus       TIINYASRVMQDNFDVQLLLLE
Candida oxycetoniae             TIINYASRVMHDNFDVQLLLLE
Hyphopichia burtonii            TILNYATRVMHDDFNVQLLLIE
Metschnikowia persimmonesis     TILNYATRVMHDNFNVELLLAE
Candida tanzawaensis            TILNYATRVMHDDFNVQLLLIE
Candida subhashii               TILNYATRVMHDDFDVQLLVME
Spathaspora passalidarum        TILNYATRVMHDDFDVQLLVME
Candida theae                   TILNYATRVMHNEFDVQLLLME
Candida margitis                TILNYATRVMHNEFDVQLLLME
Candida orthopsilosis           TILNYATRVMHNEFDVQLLLME
Candida parapsilosis            TILNYATRVMHNEFDVQLLLME
Candida auris                   TILNYATRVMHDDFNVQLLVIE
Candida haemuloni               TILNYATRVMHDDFNVQLLVIE
Yamadazyma Tenuis               TIMNYATRVMHDDFNVQLLVIE
Scheffersomyces stipitis        TILNYATRVMHDDFNVQLLVIE
Candida pseudojiufengensis      SILNYSTRVMHDDFDVQLLLLE
Candida jiufengensis            GILNYSTRVMHDDFDVQLLLLE
Scheffersomyces spartinae       LILNYATRVMHDDFNVQLLLTE
Candida jaroonii                TIINYATRVMHDDFNGQLLVIE
Candida metapsilosis            TILNYATRVMHNEFDVQLLMME
Metschnikowia bicuspidata       TVLNYATRVMHDNFNVELLIIE
Clavispora lusitaniae           TILNYATRVMHDDFNIELLVIE
Candida haemuloni               TILNYATRVMYDDFNVQLLVIE
Debaryomyces fabryi             SILNYATRVMHDDLNVQLLLME
Millerozyma farinosa            TVLNYVTRVMHDDFNVQILLAE
Debaryomyces hansenii           SILNYATRVMHNDLNVQLLLME
Candida intermedia              TILNYATRVMHDDLNVELLVME
Diutina rugosa                  TILNYATRVLHDDLNVQLLVIE
Meyerozyma guilliermondii       TILNYATRVMHDDVNMQLLVIE
                                 ::** :**: ::.: ::*: *
```

Fig. S8. Full or partial conservation of the hypothetical auto-inhibitory region of CaCdc25 in species belonging to the *Saccharomycetales* kingdom. Multiple sequence alignment of CaCdc25 (residues 837-858) with sequences of other species producing significant alignments according to BLAST (the 34 sequences with the highest score are shown). Strictly conserved alignment positions are shown in inverted type on a green background.
