# Supplementary material for: Pathogen-specific structural features of Candida albicans Ras1 activation complex: uncovering new antifungal drug targets
Source: mBio. 2023 Aug 1;14(4):e00638-23. doi: 10.1128/mbio.00638-23 (PMC10470544; doi:10.1128/mbio.00638-23)
Supplement: Supplemental methods — Text with detailed methods. [file mbio.00638-23-s0009.docx]

Supplemental methods for

Pathogen-Specific Structural Features of *C. albicans* Ras1 Activation Complex: Uncovering New Antifungal Drug Targets

José A. Manso,^a,b^# Arturo Carabias,^c^* Zsuzsa Sárkány,^a,b^ José M. de Pereda,^c^ Pedro José Barbosa Pereira,^a,b^ Sandra Macedo-Ribeiro^a,b^#

^a^IBMC – Instituto de Biologia Molecular e Celular, Universidade do Porto, Porto, Portugal

^b^Instituto de Investigação e Inovação em Saúde, Universidade do Porto, Porto, Portugal

^c^Instituto de Biología Molecular y Celular del Cáncer, Consejo Superior de Investigaciones Científicas - University of Salamanca, Salamanca, Spain

#Address correspondence to José A. Manso, [jose.manso@i3s.up.pt](mailto:jose.manso@i3s.up.pt) or Sandra Macedo-Ribeiro, [sribeiro@ibmc.up.pt](mailto:sribeiro@ibmc.up.pt).

*Present address: Arturo Carabias, Structural Molecular Biology Group, Novo Nordisk Foundation Center for Protein Research, Faculty of Health and Medical Sciences, University of Copenhagen, Copenhagen, Denmark.

**Cloning and site-directed mutagenesis**

DNA sequences encoding the full-length *C. albicans* CaRas1 (UniProt P0CY32) and the fragment comprising the first 166 amino acids were amplified from a synthetic gene by PCR and cloned into the pETGB1a expression vector using the NcoI and Acc65I restriction sites. The resulting constructs encode an N-terminal Gb1-tag, 6×His-tag and TEV cleavage site upstream the CaRas1 protein sequences. The CaRas1 fragment 1-213 (CaRas1-213) expression vector was obtained by inserting a stop codon at position N214 by site directed mutagenesis in the vector encoding the full-length protein. A sequence encoding the fragment comprising amino acids 860-1333 of CaCdc25 (UniProt P43069) was amplified from a synthetic gene by PCR and cloned into the NcoI and Acc65I restriction sites of the derivative of the pETGB1a expression vector (EMBL, Heidelberg, Germany), pETMBP_1. This construct encodes an N-terminal MBP-tag, 6×His-tag and TEV cleavage site upstream the CaCdc25 protein fragment. CaRas1 and CaCdc25 point mutations were introduced by PCR using the QuikChange method. The correctness of all constructs was verified by automated DNA sequencing (oligonucleotides used for gene amplification and site directed mutagenesis are listed below in the Supplemental Methods Tables 1 and 2, respectively).

**Protein expression and purification**

CaRas1 proteins were expressed in *Escherichia coli* BL21 (DE3). Cells were precultured at 37 °C overnight in lysogeny broth (LB) supplemented with 50 mg L^-1^ kanamycin. Twenty mL of the preculture were used to inoculate 2-L flasks containing 500 mL LB, 50 mg L^-1^ kanamycin. When cultures reached an OD_600_ of 0.6-0.8 after ~1 h shaking at 37 °C, protein production was induced by adding 900 μ*M* isopropyl-β-d-thiogalactopyranoside (IPTG) and expression proceeded for 3 h at 37 °C. Cells were harvested by centrifugation for 30 min at 4 °C and 3,500 *g* and the resulting pellets were resuspended in 20 m*M* Tris-HCl pH 7.5, 500 m*M* NaCl, 20 m*M* imidazole, 5 m*M* β-mercaptoethanol, 5 m*M* MgCl_2_, 5% (*v*/*v*) glycerol (buffer A) and frozen at -80 °C. Upon thawing, 1 m*M* PMSF, 0.2 mg mL^-1^ lysozyme, 12.5 m*M* MgCl_2_, and 10 μg mL^-1^ DNAse were added to the cell suspension and stirred for 1h on ice. The cells were then disrupted by sonication on ice (5 min at 20% amplitude), the cell lysate was centrifuged for 30 min at 4 °C and 39,200 *g* and the supernatant was loaded onto a 5-mL nickel NTA agarose column (Agarose Bead Technologies) equilibrated with buffer A. Bound protein was eluted with buffer A containing 500 m*M* imidazole. The purity of the eluted fractions was assessed by SDS-PAGE and those containing recombinant protein were pooled. The protein was dialyzed overnight at room temperature against 20 m*M* Tris-HCl pH 7.5, 150 m*M* NaCl, 5 m*M* β-mercaptoethanol, 5 m*M* MgCl_2_, 5% (*v*/*v*) glycerol in the presence of 0.1-0.5 mg of TEV (tobacco etch virus) protease fused to a 6×His-tag (His-rTEV) to remove the N-terminal MBP-tag and 6×His-tag. Pre- and post-digestion samples were analyzed by SDS-PAGE to confirm the cleavage. The His-rTEV and traces of undigested protein were removed with a second immobilized metal affinity chromatography step, in the same conditions as described above for the first. Target proteins were collected in the column flow-through, concentrated to 5 mL in a 10 kDa molecular weight cutoff centrifugal ultrafiltration device (Millipore) and loaded onto a Sephacryl S100 HR 26/60 size-exclusion chromatography column (GE Healthcare), with a mobile phase adequate to the downstream use of the protein. Fractions containing pure protein, as assessed by SDS-PAGE, were combined, concentrated by ultrafiltration, flash frozen in liquid N_2_ and stored at -80 °C, except for crystallization experiments, where the sample was used immediately. Protein concentrations were determined using the extinction coefficient at 280 nm calculated with the ProtParam tool (http://web.expasy.org/protparam/).

The expression and purification procedures for both wild type and variants of the catalytic region of CaCdc25 were as described for CaRas1, except for the expression strain (*Escherichia coli* Rosetta (DE3)), the antibiotics used (50 mg L^-1^ kanamycin and 30 mg L^-1^ chloramphenicol) and the induction conditions (200 μ*M* IPTG for overnight induction at 20 °C). Additionally, all buffers were the same as for CaRas1, except for the exclusion of 5 m*M* β-mercaptoethanol, 5 m*M* MgCl_2_ and 5% (*v*/*v*) glycerol. Since CaCdc25 precipitates on ice, all procedures with the purified protein were carried out at room temperature.

CaRas1/CaCdc25 complexes were prepared by mixing equimolar amounts of each protein in 20 m*M* sodium phosphate pH 7.5, 150 m*M* NaCl, 1m*M* EDTA, 5% (*v*/*v*) glycerol, 3 m*M* DTT (buffer B). The mixtures were incubated for 3 h at room temperature and loaded onto a Superdex 200 10/300 GL (GE Healthcare) size-exclusion chromatography column using buffer B as mobile phase (see below the Supplemental Methods Figure 1). Fractions containing both proteins, as assessed by SDS-PAGE, were pooled.

**Crystallization**

CaCdc25 crystals were obtained at 20 °C by vapor diffusion from sitting drops composed of equal volumes (1 μL) of protein solution (7.2 mg mL^-1^ in 10 m*M* Tris-HCl pH 7.5, 100 m*M* NaCl) and precipitant (23% (w/v) PEG 3350, 0.25 *M* sodium malonate dibasic monohydrate). Prior to data collection the crystals were cryoprotected in crystallization solution supplemented with 25% (*v*/*v*) glycerol and flashed-cooled in liquid N_2_.

**Data collection and processing**

A low resolution (~3 Å) diffraction dataset was collected from two isomorphous crystals of the catalytic region of CaCdc25 (dataset A, Table 1; https://doi.org/10.15785/SBGRID/860 and https://doi.org/10.15785/SBGRID/861) and used for initial structure solution. A partial model derived from dataset A was used as template for molecular replacement (see Structure solution and refinement, below) in a higher resolution (2.45 Å) dataset (dataset B, Table 1; https://doi.org/10.15785/SBGRID/859), which allowed building and refinement of the final model of CaCdc25. All diffraction data were collected at 100 K on the BL13-XALOC beamline (J. Juanhuix, F. Gil-Ortiz, G. Cuní, C. Colldelram, J. Nicolás, J. Lidón, E. Boter, C. Ruget, S. Ferrer, and J. Benach, J Synchrotron Radiat 21:678-689, 2014, https://doi.org/10.1107/s160057751400825x) of the ALBA-CELLS synchrotron (Cerdanyola del Vallès, Spain). Diffraction data were processed with XDS (W. Kabsch, Acta Crystallogr D Biol Crystallogr, 66:125-132, 2010, https://doi.org/10.1107/S0907444909047337), Pointless (P. Evans, Acta Crystallogr D Biol Crystallogr, 62:72-82, 2006, https://doi.org/10.1107/S0907444905036693), and Aimless (P. R. Evans, and G. N. Murshudov, Acta Crystallogr D Biol Crystallogr, 69:1204-1214, 2013, https://doi.org/10.1107/S0907444913000061) as implemented in the autoPROC pipeline (C. Vonrhein, C. Flensburg, P. Keller, A. Sharff, O. Smart, W. Paciorek, T. Womack, and G. Bricogne, Acta Crystallogr D Biol Crystallogr, 67:293-302, 2011, https://doi.org/10.1107/S0907444911007773). Data sets were scaled and merged with XSCALE (W. Kabsch, Acta Crystallogr D Biol Crystallogr 66:133-144, 2010, https://doi.org/10.1107/s0907444909047374). All crystals belong to the orthorhombic space group *P*2_1_2_1_2_1_ (Table 1) and contain two monomers of CaCdc25 in the asymmetric unit.

**Structure solution and refinement**

The structure of the catalytic region of CaCdc25 was solved by molecular replacement (MR) with Phaser (A. J. McCoy, R. W. Grosse-Kunstleve, P. D. Adams, M. D. Winn, L. C. Storoni and R. J. Read, J Appl Crystallogr, 40:658-674, 2007, https://doi.org/10.1107/S0021889807021206) as implemented in the MrBUMP pipeline (R. M. Keegan and M. D. Winn, Acta Crystallogr D Biol Crystallogr, 64:119-124, 2008, https://doi.org/10.1107/S0907444907037195) of the CCP4 suite (M. D. Winn, C. C. Ballard, K. D. Cowtan, E. J. Dodson, P. Emsley, P. R. Evans, R. M. Keegan, E. B. Krissinel, A. G. W. Leslie, A. McCoy, S. J. McNicholas, G. N. Murshudov, N. S. Pannu, E. A. Potterton, H. R. Powell, R. J. Read, A. Vagin, and K. S. Wilson, Acta Crystallogr D Biol Crystallogr, 67:235-242, 2011, https://doi.org/10.1107/s0907444910045749) using dataset A. An apparent solution was found using the crystal structure of the REM-CAT region of Sos1 in complex with HRas and ligands (PDB entry 4US0; J. J. G. Winter, M. Anderson, K. Blades, C. Brassington, A. L. Breeze, C. Chresta, K. Embrey, G. Fairley, et al., J Med Chem 58:2265-2274, 2015, https://doi.org/10.1021/jm501660t) as search model but after some rounds of automatic restrained refinement with REFMAC (G. N. Murshudov, P. Skubák, A. A. Lebedev, N. S. Pannu, R. A. Steiner, R. A. Nicholls, M. D. Winn, F. Long, and A. A. Vagin, Acta Crystallogr D Biol Crystallogr. 67:355-367, 2011, https://doi.org/10.1107/S0907444911001314) the value of *R*_free_ was stuck at >0.50. In order to generate other templates for MR, the initial solution from Phaser was subjected to normal mode analysis using the elNémo server (https://www.sciences.univ-nantes.fr/elnemo/; K. Suhre and Y. H. Sanejouand, Acta Crystallogr D Biol Crystallogr 60:796-799, 2004, https://doi.org/10.1107/s0907444904001982). Eleven conformations that represent global motions corresponding to the lowest-frequency mode (200 amplitude perturbation in the direction of a single normal mode with a step size of 40) were generated. Each of these conformations was used as MR search model, and although an improved MR solution was obtained with one of these models, refinement could not be improved and the value of *R*_free_ remained above 0.50. The best model was then subjected to smooth deformation with the morph module of Phenix (T. C. Terwilliger, R. J. Read, P. D. Adams, A. T. Brunger, P. V. Afonine and L-W. Hung, Acta Crystallogr D Biol Crystallogr, 69:2244-2250, 2013, https://doi.org/10.1107/S0907444913017770) and a substantial improvement in the quality of the electron density maps was observed after 2 morphing cycles (6 Å radius of morphing), accompanied by improved statistics (*R*_work_ = 0.38 and *R*_free_ = 0.48). This model was completed with alternating cycles of refinement with Phenix (P. D. Adams, P. V. Afonine, G. Bunkóczi, V. B. Chen, I. W. Davis, N. Echols, J. J. Headd, L.-W. Hung, G. J. Kapral, R. W. Grosse-Kunstleve, A. J. McCoy, N. W. Moriarty, R. Oeffner, R. J. Read, D. C. Richardson, J. S. Richardson, T. C. Terwilliger, and P. H. Zwart, Acta Crystallogr D Biol Crystallogr, 66:213-221, 2010, doi:10.1107/S0907444909052925) and manual model building with Coot (P. Emsley, B. Lohkamp, W. G. Scott, and K. Cowtan, Acta Crystallogr D Biol Crystallogr, 66:486-501, 2010, doi:10.1107/S0907444910007493). It then became evident that the relative positions of the REM and CAT domains are different in the two molecules of CaCdc25 in the asymmetric unit, probably accounting for the difficulties experienced during the phasing stage. The final refined model derived from dataset A (*R*_work_ = 0.31 and *R*_free_ = 0.34) was used as MR search model with dataset B, and further improved with iterative cycles of refinement with Phenix and model building with Coot. The final model (*R*_work_ = 0.19 and *R*_free_ = 0.24) includes residues 883-1035 and 1039-1306 of molecule A and residues 883-1034 and 1039-1305 of molecule B, with 98% of the main-chain torsion angles in the favored regions of the Ramachandran plot. Detailed refinement statistics are given in Table 1. All crystallographic software was supported by SBGrid (A. Morin, B. Eisenbraun, J. Key, P. C. Sanschagrin, M. A. Timony, M. Ottaviano, and P. Sliz, eLife, 2:e01456, 2013, https://doi.org/10.7554/elife.01456).

**Isothermal titration calorimetry (ITC)**

ITC experiments were carried out at 25 °C using a VP-ITC system (MicroCal, Northampton, MA, USA). Two solutions of 21 and 42 μ*M* CaCdc25 (in 20 m*M* sodium phosphate pH 7.4, 250 m*M* NaCl, 5 m*M* β-mercaptoethanol, 5 m*M* MgCl_2_, 5% (*v*/*v*) glycerol) were titrated with solutions of 482 μ*M* CaRas1-FL and 370 μ*M* CaRas1-166, respectively, in the same buffer as the CaCdc25 sample and incubated with 10 m*M* EDTA for 30 min prior to loading in the injection syringe. Titrations were done with one initial injection of 3 μL followed by 13 sequential injections of 20 μL each, with 15 μcal s^-1^ reference power, 307 rpm stirring speed and 240 s interval. Heat exchange from the first injection was not used in the analysis. Data were analyzed using the Origin 7 software package (MicroCal) and corrected by the heat of injection calculated from the basal heat remaining after saturation and confirmed by titration into buffer only as control. A single-site model was applied to obtain the stoichiometry (*N*), and association constant (*K*_a_) using a nonlinear squares algorithm.

**Circular dichroism measurements**

CaRas1 proteins were dissolved in 10 m*M* sodium phosphate pH 7.5, 100 m*M* NaF, 0.5 m*M* MgCl_2_, 5% (*v*/*v*) glycerol or in the same buffer with 50% (*v*/*v*) TFE, to a final concentration of 0.1 mg mL^-1^. CD spectra (190-240 nm spectral range) were recorded using a Jasco J-815 CD spectrometer in a 0.1 cm quartz cuvette for far-UV CD spectroscopy.

**In vitro nucleotide exchange activity assay**

Guanine nucleotide exchange activity was measured by following changes in the fluorescence of the GDP derivative mant-dGDP [2’-Deoxy-3’-O-(N-Methyl-anthraniloyl) guanosine-5’-diphosphate sodium salt (Jena Bioscience GmbH); S. M. Margarit, H. Sondermann, B. E. Hall, B. Nagar, A. Hoelz, M. Pirruccello, D. Bar-Sagi, and J. Kuriyan, Cell 112:685-695, 2003, <https://doi.org/10.1016/s0092-8674(03)00149-1>; H. Rehmann, Methods Enzymol 407:159-173, 2006, https://doi.org/10.1016/s0076-6879(05)07014-x]. CaRas1 proteins (200 μ*M* in 20 m*M* Tris-HCl pH 7.5, 50 m*M* NaCl, 4 m*M* EDTA, 1m*M* DTT) were loaded by incubation with 2 m*M* mant-dGDP for 1.5 h at 4 °C (200 μL final volume). The loading reaction was stopped by addition of 10 m*M* MgCl_2_ (30 min, 4 °C). Precipitate formed by addition of MgCl_2_ was removed by centrifuging the sample at 16,000 *g* (30 min, 4 °C). Excess nucleotide was removed by SEC on a Superdex 200 (10/300) column pre-equilibrated with 20 m*M* Tris-HCl pH 7.5, 50 m*M* NaCl, 10 m*M* MgCl_2_. Protein-containing fractions were pooled and concentrated using centrifugal ultra-filtration devices (10 kDa cut-off; Millipore). Protein concentration was determined using a Bradford assay with a standard calibration curve with BSA. Fluorescence measurements were performed on a Fluoromax-4 spectrofluorometer (Horiba-Jobin Ybon), with excitation at 355 nm (1 nm bandwidth) and emission at 450 nm (10 nm bandwidth). Nucleotide exchange experiments were performed at 25 °C in 50 m*M* Tris-HCl pH 7.5, 150 m*M* NaCl, 5 m*M* MgCl_2_. A typical exchange reaction mix contained 200 n*M* CaRas1-mant-dGDP and the reaction was started by addition of 100 n*M* CaCdc25 and 40 μ*M* unlabeled GDP (200-fold molar excess with respect to CaRas1). The exchange rate constant (*k*_obs_) was determined by fitting a single exponential decay model to the time-dependent decrease of fluorescence intensity. Statistical analysis was performed by one-way analysis of variance (ANOVA) followed by Dunnett’s multiple comparisons test with Prism 8 (GraphPad Software).

A peptide corresponding to the region 837–858 (TIINYATRVMQDNFDVQLLLVE) of CaCdc25 was custom synthesized (Genosphere Biotechnologies, France). The effect of this synthetic peptide (at a concentration range of 1-80 μ*M*) on nucleotide exchange assays was assessed as described above using 50 n*M* CaCdc25. To evaluate the effect of peptide 837-858, the apparent exchange rate constant (*k*_app_) was determined by fitting a double exponential model to the data, which is defined by the sum of factors a_1_*k*_1_ and a_2_*k*_2_ (where a_1_ and a_2_ are the amplitudes of the first and second exponential, respectively, and *k*_1_ and *k*_2,_ the corresponding rate constants). EC50 value was calculated by fitting the dose-dependent *k*_app_ data to the Hill model with SigmaPlot (Systat Software). Two control peptides with the same amino acid composition as peptide 837–858 but with the scrambled amino acid sequences NDEVLLADQLVFVTQTIMYRNI (SCR-1) and QYRITDLVNLDLQEVFVTMINA (SCR-2) were also custom synthesized (Genosphere Biotechnologies, France). SCR-1 was insoluble in the reaction buffer and only the effect of SCR-2 on the nucleotide exchange activity of CaCdc25 could be evaluated (see Supplemental Methods Figure 2 below).

**SAXS measurements and data analysis**

SAXS data were measured at the P12 beamline of the European Molecular Biology Laboratory (EMBL) at the Deutsches Elektronen-Synchrotron (DESY; Hamburg, Germany) using radiation of 1.24 Å wavelength and a Pilatus 6M detector (Dectris) (C. E. Blanchet, A. Spilotros, F. Schwemmer, M. A. Graewert, A. Kikhney, C. M. Jeffries, D. Franke, D. Mark, R. Zengerle, F. Cipriani, S. Fiedler, M. Roessle, and D. I. Svergun, J Appl Crystallogr 48:431-443, 2015, https://doi.org/10.1107/s160057671500254x). Samples were equilibrated in adequate buffers (Table S1), concentrated by ultrafiltration (on 10 kDa or 30 kDa molecular weight cut-off centrifugal devices; Millipore), and centrifuged at 21,500 g and 4 °C for 30 min to remove any aggregates. Prior to data collection, thawed samples were centrifuged and solutions of various concentrations, in the range of 1.1-31.0 mg mL^-1^, were prepared by 2-fold serial dilutions in order to evaluate the magnitude of interparticle effects. All samples and their corresponding buffers were measured consecutively in standard "batch" mode using an automated sample changer, which ensures continuous flow. Thirty frames (0.1 s exposure) for each protein/buffer sample were collected over a 0.0033 to 0.727 Å^-1^ (*q* = (4π sin*θ*)/*λ*, where 2*θ* is the scattering angle) scattering vector, at 10 °C (20 °C for CaCdc25 samples). Data were processed and analyzed with the ATSAS 3.0 package (K. Manalastas-Cantos, P. V. Konarev, N. R. Hajizadeh, A. G. Kikhney, M. V. Petoukhov, D. S. Molodenskiy, A. Panjkovich, H. D. T. Mertens, A. Gruzinov, C. Borges, C. M. Jeffries, D. I. Svergun, and D. Franke, J Appl Crystallogr 54:343-355, 2021, https://doi.org/10.1107/s1600576720013412). Extrapolation from multiple scattering curves at different concentrations to a zero-concentration curve and Guinier analysis were done with PRIMUS/qt (P. V. Konarev, V. V. Volkov, A. V. Sokolova, M. H. J. Koch, and D. I. Svergun. J Appl Crystallogr, 36:1277-1282, 2003, https://doi.org/10.1107/S0021889803012779). The radius of gyration (*R*_g_) for CaRas1 samples remained constant for all concentrations, within the experimental error. Although a clear dependence of *R*_g_ with concentration was observed for samples of CaCdc25 and of CaRas1/CaCdc25 complexes, extrapolation to zero concentration was used to alleviate the possible influence of interparticle interactions. The pair distance distribution function, *P*(*r*), was calculated with GNOM (D. I. Svergun, J Appl Crystallogr, 25:495-503, 1992, https://doi.org/10.1107/S0021889892001663) and *ab initio* shape reconstructions were calculated with DAMMIF (D. Franke, and D. I. Svergun, J Appl Cryst 42:342-346, 2009, https://doi.org/10.1107/s0021889809000338); multiple reconstructions were superimposed, averaged, and filtered with DAMAVER (V. V. Volkov, and D. I. Svergun, J Appl Cryst 36:860-864, 2003, https://doi.org/10.1107/S0021889803000268). Flexibility of the CaRas1 hypervariable region was evaluated using EOM (P. Bernadó, E. Mylonas, M. V. Petoukhov, M. Blackledge, and D. I. Svergun, J Am Chem Soc 129:5656-5664, 2007, https://doi.org/10.1021/ja069124n). The scattering profiles of atomic structures were calculated with CRYSOL (D. Svergun, C. Barberato, and M. H J. Koch, J Appl Crystallogr, 28:768-773, 1995, https://doi.org/10.1107/S0021889895007047) and missing sequence regions were modeled with MODELLER (B. Webb, and A. Sali, Curr Protoc Bioinformatics 47:5.6.1-5.6.32, 2014, https://doi.org/10.1002/0471250953.bi0506s15).

**Sequence and atomic structures analysis**

Evolutionary conservation scores were calculated with ConSurf ([https://consurf.tau.ac.il/](https://consurf.tau.ac.il/)); H. Ashkenazy, S. Abadi, E. Martz, O. Chay, I. Mayrose, T. Pupko, and N. Ben-Tal, Nucleic Acids Res, 44:W344-W350, 2016, https://doi.org/10.1093/nar/gkw408). Atomic structures were superposed with Theseus (D. L. Theobald, and P. A. Steindel, Bioinformatics 28:1972-1979, 2012, https://doi.org/10.1093/bioinformatics/bts243) and represented with PyMOL v. 1.6.0 (Schrödinger) and the secondary structure of CaCdc25 was assigned with DSSP (W. Kabsch, and C. Sander, Biopolymers 22:2577-2637, 1983, https://doi.org/10.1002/bip.360221211).

**Generation of AlphaFold2 models**

Predicted models for full-length CaRas1, isolated and complexed with the catalytic region of CaCdc25, the complex CaCdc25/G-domain, and for CaCdc25 were generated by the AI-based tools AlphaFold2 and AlphaFold2-multimer using MMseqs2 through the ColabFold notebook (M. Mirdita, K. Schütze, Y. Moriwaki, L. Heo, S. Ovchinnikov, and M. Steinegger, Nat Methods 19:679-682, 2022, https://doi.org/10.1038/s41592-022-01488-1). Five models were generated for each protein or protein complex using the pdb70 database as template.

**Supplemental Methods Table 1. Oligonucleotides used to amplify CaRas1 and the GEF catalytic region of CaCdc25 ORFs.**

| **Primer name** | **Sequence (5’ - 3’)** |
| --- | --- |
| Ras1-166 Forward | CCGGCCATGGCGCTGCGTGAATACAAAC  NcoI A L R E Y K |
| Ras1-166 Reverse | CCGGGGTACC*TTA*GTTGATGTTGCGCACCAG  Acc65I N I N R V L |
| Ras1-Full Reverse | CCGGGGTACC*TTA*TACAATGAC  Acc65I V I V |
| GEF Forward | CCGGCCATGGGCAATAATACGAGTT  NcoI G N N T S |
| GEF Reverse | CCGGGGTACC*TTA*TTTCAGCGAGAAC  Acc65I K L S F |

**Supplemental Methods Table 2. Oligonucleotides used for site-directed mutagenesis of CaRas1 and of GEF catalytic region of CaCdc25.**

| **Primer name** | **Sequence (5’ - 3’)** |
| --- | --- |
| Ras1-213 Forward | CCAAATCAATAAC***T****A****G***AACAACACTTCTGCAGTCAATGGC  Stop |
| Ras1-213 Reverse | GCCATTGACTGCAGAAGTGTTGTT***C****T****A***GTTATTGATTTGG  Stop |
| GEF-H1234E Forward | GACCTTCGTCTAC**G**A**A**GGCAACCCGGACTATC  E |
| GEF-H1234E Reverse | GATAGTCCGGGTTGCC**T**T**C**GTAGACGAAGGTC  E |
| GEF-H1234D Forward | GACCTTCGTCTAC**G**ACGGCAACCCGGACTATC  D |
| GEF-H1234D Reverse | GATAGTCCGGGTTGCCGT**C**GTAGACGAAGGTC  D |
| GEF-H1234A Forward | GACCTTCGTCTAC**GC**CGGCAACCCGGACTATC  A |
| GEF-H1234A Reverse | GATAGTCCGGGTTGCCG**GC**GTAGACGAAGGTC  A |

**
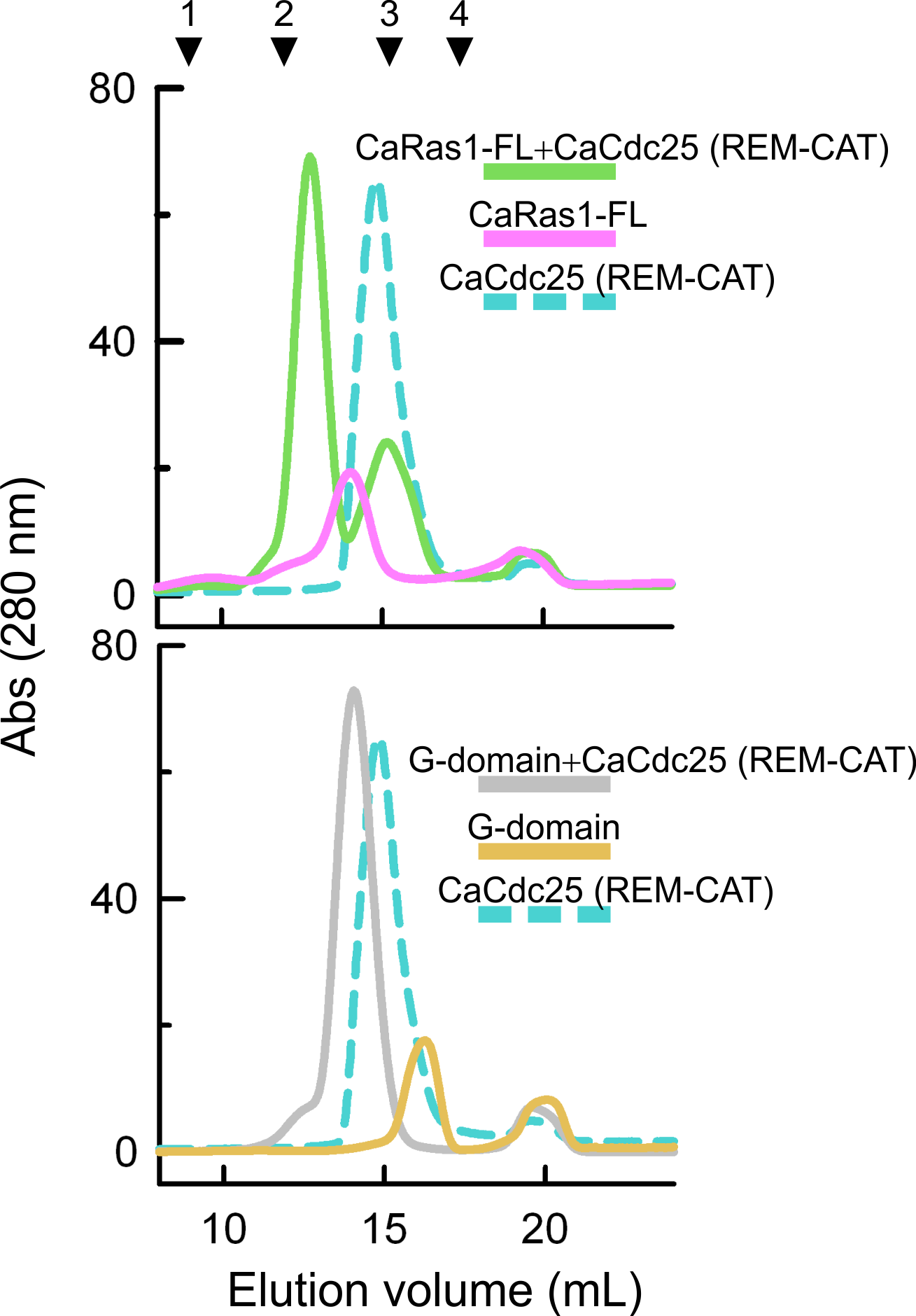
**

**Supplemental Methods Figure 1.** Analysis by size exclusion chromatography of the interaction of the catalytic region of CaCdc25 (REM-CAT) with CaRas1-FL and CaRas1 G-domain. CaRas1/REM-CAT complexes were prepared by mixing equimolar amounts of each protein in 20 m*M* sodium phosphate pH 7.5, 150 m*M* NaCl, 1mM EDTA, 5% (*v*/*v*) glycerol, 3 m*M* DTT. The mixture was incubated for 3 h at room temperature and loaded onto a Superdex 200 10/300 GL (GE Healthcare) size-exclusion chromatography column using the above buffer as mobile phase. Chromatograms of the isolated proteins (REM-CAT (blue), CaRas1-FL (pink) and CaRas1 G-domain (orange)) and of the equimolar mixtures of the catalytic region of CaCdc25 with CaRas1-FL (green) and CaRas1 G-domain (grey) are shown. The inverted triangles above the chromatograms mark the position of the elution peaks of the proteins used as standards: (1) thyroglobulin (670 kDa), (2) γ-globulin (158 kDa), (3) ovalbumin (44 kDa), and (4) myoglobin (17 kDa).


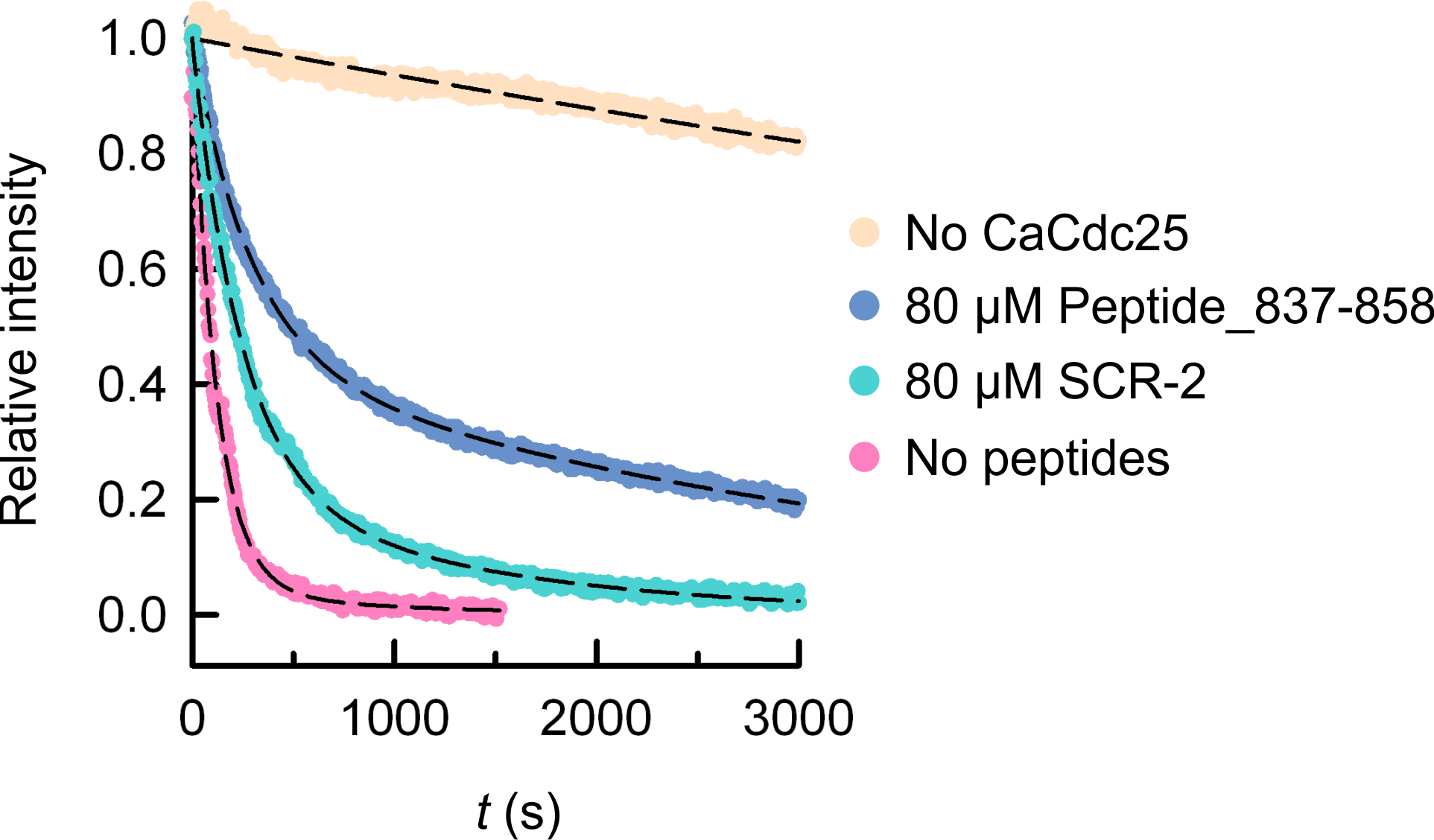


**Supplemental Methods Figure 2.** Effect of peptides on the catalytic activity of CaCdc25. (A) Nucleotide exchange reactions of CaRas1-mant-dGDP (200 n*M*) catalyzed by CaCdc25 (50 n*M*) in the presence of peptide 837-858 (80 µ*M*) or of the scrambled peptide SCR-2 with the same amino acid composition as peptide 837-858 (80 μ*M*). Under the same reaction conditions and at the same concentration, the SCR-2 peptide displayed a much lower impact in the CaCdc25 activity than peptide 837-858 (*k*_app_ (peptide 837-858) / *k*_app_ (SCR-2) = ~0.5. The SCR-2 inhibitory effect is likely due to unspecific interactions with CaCdc25, driven mostly by its hydrophobic nature (~50% hydrophobic amino acid content) and that of the binding surface in CaCdc25 for the G-domain of CaRas1, which may be exacerbated by the 3 order of magnitude molar excess of the peptide relative to CaCdc25.
