# Supplementary material for: Pathogen-specific structural features of Candida albicans Ras1 activation complex: uncovering new antifungal drug targets
Source: mBio. 2023 Aug 1;14(4):e00638-23. doi: 10.1128/mbio.00638-23 (PMC10470544; doi:10.1128/mbio.00638-23)
Supplement: Table S1 — Small angle X-ray scattering results for CaCdc25 (tandem REM-CAT), CaRas1 constructs and CaRas1/CaCdc25 complexes. [file mbio.00638-23-s0010.docx]

**Table S1. Small angle X-ray scattering results for CaCdc25 (tandem REM-CAT), CaRas1 constructs and CaRas1/CaCdc25 complexes*^a^*.**

| **Sample details** | **CaCdc25 (REM-CAT)** | **CaRas1 G-domain** | **CaRas1-FL** | **CaRas1 G-domain/CaCdc25** | **CaRas1-FL/CaCdc25** |
| --- | --- | --- | --- | --- | --- |
| Organism | *Candida albicans* | | | | |
| Source | Expressed in *E. coli* BL21 (DE3) Rosetta | Expressed in *E. coli* BL21 (DE3) | | As described for the individual components | |
| UniProtKB entry (residues in construct) | P43069 (860-1333) | P0CY32 (1-166) | P0CY32 (1-290) | As described for the individual components | |
| $\bar{\nu}$ from chemical composition (cm^3^ g^-1^)*^b^* | 0.738 | 0.733 | 0.719 | 0.737 | 0.731 |
| Particle contrast from sequence and  solvent constituents,  Δ$\bar{\rho}$ (*ρ*_protein_ - *ρ*_solvent_; 10^10^ cm^-2^)*^b^* | 2.70 (12.29 − 9.59) | 2.77 (12.35 − 9.58) | 3.00 (12.58 −9.58) | 2.72 (12.31 − 9.59) | 2.81 (12.40 − 9.59) |
| Concentration range (mg mL^-1^) | 1.4 − 11.1 | 1.9 − 31.0 | 2.1 − 17.0 | 1.1 − 8.5 | 1.4 − 11.3 |
| *M* from chemical composition (kDa) | 55 | 19 | 33 | 74 | 87 |
| Solvent (solvent blanks taken from SEC  flow-through prior to elution of protein) | 20 m*M* sodium phosphate pH 7.5, 150 m*M* NaCl, 5% (v/v) glycerol, 3 m*M* DTT | 20 m*M* Tris-HCl pH 7.5, 150 m*M* NaCl, 5% (v/v) glycerol, 3 m*M* DTT, 5 m*M* MgCl_2_ | | 20 m*M* sodium phosphate pH 7.5, 150 m*M* NaCl, 1m*M* EDTA, 5% (v/v) glycerol, 3 m*M* DTT | |
| **Structural parameters** |  |  | |  | |
| Guinier analysis |  |  |  |  |  |
| *I*(0)/c (10^-2^ cm^2^ mg^-1^)*^c^* | 3.21 ± 0.01 | 1.01 ± 0.01 | 1.89 ± 0.01 | 4.13 ± 0.01 | 6.15 ± 0.01 |
| *R*_g_ (Å) | 31.7 ± 0.1 | 16.1 ± 0.1 | 36.2 ± 0.1 | 32.0 ± 0.6 | 39.7 ± 0.1 |
| *q*_min_ (Å^-1^) | 0.012 | 0.042 | 0.022 | 0.029 | 0.014 |
| *qR*_g_ max | 1.30 | 1.30 | 1.30 | 1.30 | 1.18 |
| Correlation coefficient, *R*^2^ | 0.9991 | 0.9995 | 0.9928 | 0.9961 | 0.9976 |
| *M* from bayesian inference [Credibility  interval probability] (ratio to predicted) | 58 [91%] (1.1) | 17 [93%] (0.9) | 37 [93%] (1.1) | 53 [93%] (0.7) | 94 [91%] (1.1) |
| *M* from *I*(0)/c (ratio to predicted)*^d^* | 43 (0.8) | 11 (0.6) | 21 (0.6) | 54 (0.7) | 77 (0.9) |
| *M* from BSA (ratio to predicted) | 43 (0.8) | 14 (0.7) | 25 (0.8) | 55 (0.7) | 85 (1.0) |
| *P*(*r*) analysis |  |  |  |  |  |
| *I*(0)/c (10^-2^ cm^2^ mg^-1^) *^c^* | 3.25 ± 0.01 | 1.02 ± 0.01 | 1.99 ± 0.01 | 4.17 ± 0.01 | 6.28 ± 0.01 |
| *R*_g_ (Å) | 32.9 ± 0.1 | 16.0 ± 0.1 | 41.1 ± 0.1 | 33.1 ± 0.6 | 42.8 ± 0.1 |
| *d*_max_ (Å) | 118 | 41 | 151 | 118 | 177 |
| *q* range (Å^-1^) | 0.012 − 0.300 | 0.042 − 0.497 | 0.020 − 0.34 | 0.021 − 0.25 | 0.014 − 0.20 |
| Total estimate from *GNOM* | 0.73 | 0.62 | 0.59 | 0.82 | 0.71 |
| *M* from *I*(0) (ratio to predicted value)*^d^* | 44 (0.8) | 11 (0.6) | 22 (0.7) | 55 (0.7) | 79 (0.9) |
| Porod volume (Å^3^)  (ratio *V*_P_ / *M* from sequence) | 81,103.9 (1.5) | 26,029.1 (1.4) | 55,522.3 (1.7) | 99,438.5 (1.3) | 148,691 (1.7) |

**Table S1.** Continuation.

| **Shape model-fitting results** | **CaCdc25 (REM-CAT)** | **G-domain** | **CaRas1-FL** | **CaRas1 G-domain/CaCdc25** | **CaRas1-FL/CaCdc25** |
| --- | --- | --- | --- | --- | --- |
| DAMMIF (interactive mode, 15 calculations) |  |  |  |  |  |
| *q* range for fitting (Å^-1^) | 0.012 − 0.300 | 0.042 − 0.497 | 0.020 − 0.34 | 0.021 − 0.25 | 0.014 − 0.20 |
| Symmetry, anisotropy assumptions | *P*1, prolate | *P*1, unknown | *P*1, prolate | *P*1, prolate | *P*1, prolate |
| NSD (standard deviation), No. of clusters | 1.05 (0.08), 1 | 0.83 (0.04), 1 | 0.75 (0.05), 1 | 0.89 (0.12), 1 | 0.93 (0.06), 1 |
| *χ*^2^ range | 1.49 − 1.53 | 3.57 − 3.62 | 1.93 − 2.00 | 1.69 − 1.72 | 1.49 − 1.55 |
| Constant adjustment to intensities | 0.235 | Skipped, unable to determine | Skipped, unable to determine | 2.06 | 1.47 |
| Resolution (from SASRES) (Å) | 42 ± 3 | 24 ± 2 | 29 ± 2 | 39 ± 3 | 47 ± 4 |
| *M* estimate as 0.5 × volume of models  (kDa) (ratio to expected) | 52 (0.9) | 10 (0.5) | 29 (0.9) | 65 (0.9) | 98 (1.1) |
| **Atomistic modelling** |  |  |  |  |  |
| Crystal structures | 7NZZ (This work) | Structure modelled using 2ERY as template | Structure modelled using AlphaFold2 | Structure modelled using AlphaFold2 | Structure modelled using AlphaFold2 |
| *q* range for all modelling | 0.0098 − 0.50 | 0.0395 − 0.65 | 0.0046 − 0.50 | 0.0243 − 0.30 | 0.0082 − 0.30 |
| *CRYSOL* (max. order of harmonics 50) |  |  |  |  |  |
| Constant subtraction allowed |  |  |  |  |  |
| *χ^2^* | 2.86 | 2.71 | 33.90*^e^* | 7.09*^e^* | 11.54*^e^* |
| Predicted *R*_g_ (Å) | 31.8 | 16.1 | 37.9 | 31.8 | 46.8 |
| Vol (Å), Ra (Å), Dro (e Å^-3^) | 72,996 / 1.80/ 0.048 | 24,269 / 1.40 / 0.005 | 36,356 / 1.74 / 0.000 | 87,726 / 1.80 / 0.005 | 100,139 / 1.40 / 0.050 |
| No constant subtraction |  |  |  |  |  |
| *χ^2^* | 2.92 | 4.49 | 39.74*^e^* | 25.97*^e^* | 12.04*^e^* |
| Predicted *R*_g_ (Å) | 31.8 | 16.3 | 38.0 | 31.5 | 46.9 |
| Vol (Å), Ra (Å), Dro (e Å^-3^) | 71,958 / 1.74 / 0.052 | 22,738 / 1.44 / 0.015 | 36,356 / 1.40 / 0.003 | 99,268 / 1.80 / 0.000 | 105,552 / 1.40 / 0.037 |
| *EOM* (default parameters, 10 000 models in initial ensemble, native-like models, constant subtraction allowed) |  |  |  |  |  |
| Minimum number of curves per ensemble |  |  | 5 |  | 5 |
| Curve repetition in the ensemble allowed? |  |  | Yes |  | Yes |
| *χ*^2^ |  |  | 2.49 |  | 2.55 |
| *R*flex(random)/*R*sigma |  |  | ~73.3% (~84.1%)/0.61 |  | ~74.6% (~88.3%)/0.55 |
| Constant subtraction |  |  | 0.182 |  | 0.424 |
| No. of representative structures |  |  | 5 |  | 4 |
| **SASBDB code*^f^*** | SASDM75 | SASDM55 | SASDM65 | SASDM85 | SASDM95 |

**Table S1.** Continuation.

*^a^*Description of the accuracy and confidence in the SAXS data and modelling outputs are reported following the *2017 publication guidelines and recommendations for solution small-angle scattering data* (J. Trewhella, A. P. Duff, D. Durand, F. Gabel, J. M. Guss, W. A. Hendrickson, et al., Acta Crystallogr D Biol Crystallogr 73:710-728, 2017, https://doi.org/10.1107/s2059798317011597). *^b^*Partial specific volumes, $\bar{\nu}$ , and the particles contrast, Δ$\bar{\rho}$, were calculated with MULCh (A. E. Whitten, S. Cai, and J. Trewhella, J Appl Cryst 41:222-226, 2008, https://doi.org/10.1107/S0021889807055136). *^c^*Absolute intensities were determined using water as secondary standard. *^d^M* was calculated as [*N*_A_ *I*(0)/*c*]/Δ*ρ*_M_^2^, where *I*(0)/*c* is the forward scattering normalized against concentration, Δ*ρ*_M_ = [*ρ*_M,prot_ - (*ρ*_solv_ $\bar{\nu}$)]*r*_o_ is the scattering contrast per mass, *N*_A_ = 6.023 × 10^23^ mol^-1^ is the Avogadro number, *ρ*_M,prot_ = 3.22 × 10^23^ e g^-1^ is the number of electrons per mass of dry protein, *ρ*_solv_ = 3.34 × 10^23^ e cm^-3^ is the number of electrons per volume of the aqueous solvent, $\bar{\nu}$ is the partial specific volume of the protein and *r*_o_ = 2.8179 × 10^-13^ cm is the scattering length of an electron (L. A. Feigin, and D. I. Svergun, *in* G. W. Taylor, ed., Structure Analysis by Small-Angle X-Ray and Neutron Scattering, 1st ed., 1987, <https://doi.org/10.1007/978-1-4757-6624-0>; E. Mylonas, and D. I. Svergun, J Appl Cryst 40:s245-s249, 2007, <https://doi.org/10.1107/S002188980700252X>; D. Orthaber, A. Bergmann, and O. Glatter, J Appl Cryst 33:218-225, 2000, https://dx.doi.org/10.1107/S0021889899015216). *^e^*The lowest value of *χ*^2^ of the five generated models was selected. *^f^*SASBDB, Small Angle Scattering Biological Data Bank (E. Valentini, A. G. Kikhney, G. Previtaly, C. M. Jeffries, and D. I. Svergun, Nucleic Acids Res 43:D357-D363, https://doi.org/10.1093%2Fnar%2Fgku1047).
